# Salt‐Responsive Switchable Block Copolymer Brushes with Antibacterial and Antifouling Properties

**DOI:** 10.1002/mabi.202400261

**Published:** 2024-11-27

**Authors:** Rafael Methling, Michael Greiter, Jiwar Al‐Zawity, Mareike Müller, Holger Schönherr, Dirk Kuckling

**Affiliations:** ^1^ Department of Chemistry Paderborn University Warburger Str. 100 33098 Paderborn Germany; ^2^ Physical Chemistry I Research Center of Micro and Nanochemistry and (Bio)Technology (***Cμ***) University of Siegen Adolf‐Reichwein‐Str. 2 57076 Siegen Germany

**Keywords:** antibacterial coatings, antipolyelectrolyte effect, salt switchable polymers, zwitterionic brushes

## Abstract

A strategy for multifunctional biosurfaces exploiting multiblock copolymers and the antipolyelectrolyte effect is reported. Combining a polyzwitterionic/antifouling and a polycationic/antibacterial block with a central anchoring block for attachment to titanium oxide surfaces affords surface coatings that exhibit antifouling properties against proteins and allow for surface regeneration by clearing adhering proteins by employing a salt washing step. The surfaces also kill bacteria by contact killing, which is aided by a nonfouling block. The synthesis of block copolymers of 4‐vinyl pyridine (VP), dimethyl 4‐vinylbenzyl phosphonate (DMVBP), and 4‐vinylbenzyltrimethyl ammonium chloride (TMA) is achieved on the multigram scale via RAFT polymerization with good end group retention and narrow dispersities. By polymer analogous reactions, poly(4‐vinyl pyridinium propane sulfonate‐*block*‐4‐vinylbenzyl phosphonic acid‐*block*‐4‐vinylbenzyl trimethylammonium chloride) (P(VSP_64_‐*b*‐PA_14_‐*b*‐TMA_64_)) is obtained. The antifouling properties against the model protein pepsin and the salt‐induced surface regeneration are shown in surface plasmon resonance (SPR) experiments, while independently the antibacterial and antifouling properties of coated titanium substrates are successfully tested in preliminary microbiological assays against *Staphylococcus aureus* (*S. aureus*) and *Escherichia coli* (*E. coli*). This strategy may contribute to the development of long‐term effective antibacterial implant surface coatings to suppress biomedical device‐associated infections.

## Graphical abstract

A strategy for multifunctional antibacterial surfaces exploiting multiblock copolymers and the antipolyelectrolyte effect for surface regeneration is reported. Combining a polyzwitterionic/antifouling and a polycationic/antibacterial block with a central anchoring block for attached to titanium oxide surfaces affords surface coatings that kill bacteria by contact killing and that can be cleared from adhering proteins by employing a salt washing step.

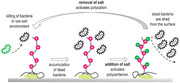

## 1 Introduction

The suppression of the attachment of bacteria on implant surfaces represents an area of significant importance since biomedical device‐associated infections (BAIs) are considered to be among the leading causes for implant failure.^[^ ^1^, ^2^, ^3^, ^4^, ^5^ ^]^ After implantation, the processes of adverse bacterial biofilm colonization and the targeted tissue integration compete with each other.^[^ ^6^ ^]^ Since the immune response in affected patients may be compromised, e.g., by the tissue's foreign body response, bacteria may survive on and nearby the implant, leading to infection and the necessity to remove the implant, unless the infection can be controlled. Ideally, the implant should actively suppress the attachment of planktonic bacteria on the implant surface, because in case remaining bacteria colonize the implant the formation of bacterial biofilm could make the necessary antibiotic treatment much more challenging.^[^ ^7^, ^8^ ^]^

To tackle this challenge a multitude of approaches has been reported. These range from surface passivating to drug releasing surface coatings on metallic implants derived, e.g., from titanium.^[^ ^9^, ^10^, ^11^ ^]^ For instance, low‐ or antifouling surface modifications have been proposed to prevent nonspecific adsorption of (bio)molecules on implants.^[^ ^12^ ^]^ Such systems exploit the pronounced hydration layer surrounding surface‐tethered molecules, impeding the adhesion of solution‐borne molecules as well as bacteria.^[^ ^13^, ^14^ ^]^ Poly(ethylene glycol) (PEG) and other hydrophilic materials bind water molecules via hydrogen bonding and thus prevent the close contact and attachment of larger molecules as they cannot penetrate the dense hydration shell. An even stronger hydration shell can be formed by moieties which induce order through electrostatic interactions, namely zwitterionic compounds.^[^ ^12^ ^]^

However, non‐regenerating antibacterial surfaces may lose their efficacy due to a shielding effect by bacterial debris of killed bacteria.^[^ ^15^ ^]^ Similarly, drug‐releasing surfaces are only active until the antibacterial compound is consumed.^[^ ^7^, ^16^, ^17^ ^]^ Once a biofilm has formed on a surface, the extracellular polymeric matrix protects the incorporated bacteria. This leads to the necessity to use higher concentrations of conventional antibiotics to treat the infection. Thus, the eradication of bacterial pathogens becomes extremely difficult. To address the loss in activity,^[^ ^8^ ^]^ there have been advances in the use of degradable multilayer systems which regenerate through a shedding mechanism, still, the lifetime is limited by the number of layers.^[^ ^18^ ^]^

Inspired by research on antifouling surfaces, the synergy of antibacterial/polycationic and antiadhesive/polyzwitterionic materials and coatings has gained some attention in recent years.^[^ ^19^, ^20^, ^21^ ^]^ Responsive systems that allow one to switch between both bacterial killing and desorption of dead bacteria and debris seem particularly sustainable and are promising candidates for future implant systems.^[^ ^22^, ^23^, ^24^ ^]^ Among the requirements for such systems are in addition to the ease of preparation, the incorporation of various functionalities: a stable cytocompatible surface coating on TiO_2_, efficient contact killing of bacteria, response to an accessible stimulus to switch the functionality to a state in which adhering biomaterial, such as bacterial debris, can desorb, followed by a reactivation.

Block copolymers allow one to combine the necessary functions in one molecule. The design of the blocks can be based on the vast knowledge of polymers for the envisioned functions. Despite the diversity of antimicrobial polymers, several main characteristics have been identified: virtually every antimicrobial polymer comprises spatially organized hydrophobic and cationic groups.^[^ ^25^, ^26^, ^27^ ^]^ In synthetic antimicrobial polymers the use of different cationic moieties, e.g., phosphonium salts,^[^ ^28^ ^]^ protonated amines,^[^ ^29^ ^]^ guanidinium,^[^ ^30^ ^]^ and alkylated pyridinium^[^ ^31^ ^]^ was explored on various types of backbones. Beyond the fact that the polymer should contain cationic and hydrophobic groups, an absolute rule for designing the perfect material has not been found yet and the efficacy remains highly specific for the combinations of an antibacterial polymer and the tested bacterial strains.^[^ ^30^, ^32^ ^]^

In seminal works, Tiller et al. modified glass slides with linear pyridinium‐based polyelectrolytes in order to transfer the bactericidal action of polycations from solution to surfaces.^[^ ^33^, ^34^ ^]^ These authors exposed the coated glass to different bacterial suspensions that have been sprayed and dried on the coated glass and showed that up to 99% of the deposited bacteria were killed. These results provided a working hypothesis, which spawned a tremendous amount of research in this field.^[^ ^35^, ^36^, ^37^, ^38^, ^39^ ^]^ It also identified structural parameters like charge density as crucial for the interactions between surface‐bound polymers and bacteria.

In the approach proposed here, polyzwitterionic moieties are combined with an antibacterial contact killing moieties such that switching between the two states should become possible. In addition, a stable anchoring to implant surfaces is also required. This work is further motivated by reports on the combination of polycationic and polyzwitterionic moieties by block copolymerization or grafting of antifouling polymers onto antimicrobial networks.^[^ ^39^, ^40^ ^]^ Both bactericidal effect and decreased fouling were detected, which offers an improvement compared to purely contact killing surfaces.

Polycations (as well as polyanions) exhibit additionally a characteristic salt dependent behavior in water that has been termed “polyelectrolyte effect.”^[^ ^41^ ^]^ The remaining class of charged polymers, polyzwitterions, shows the opposing behavior in aqueous solution. In the absence of electrolyte, the strong electrostatic forces between intermolecular and intramolecular chains lead to a collapse of the polymer into an insoluble solid.^[^ ^41^ ^]^ Upon addition of salt, however, the charges between the zwitterionic moieties are shielded and allow the chains to extend into solution, which is referred to as “antipolyelectrolyte effect.” The concentration required to dissolve a specific amount of polyzwitterionic polymer is polymer specific and depends on the concentration as well as the type and charge of the ions.^[^ ^42^ ^]^

**Figure 1:**
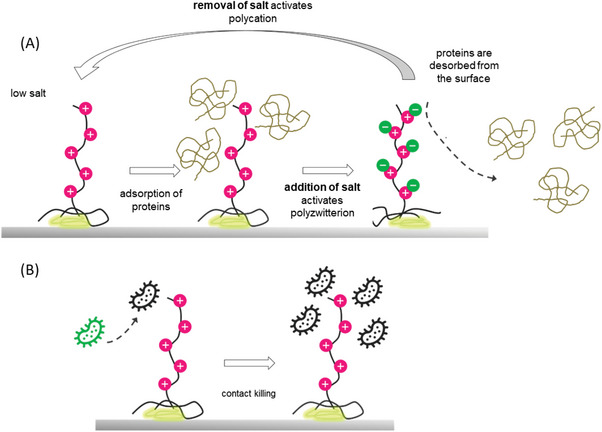
Schematic representation of the envisioned multifunctional surface. A) The salt‐responsive triblock copolymer on a titanium surface with a phosphonic‐acid‐anchor segment (yellow) and cationic/zwitterionic bristles allows for shedding of proteins that may adsorb on the surface in low salt conditions. By exposing the surface to a solution of high ionic strength, the polyzwitterionic functionality is activated. The extremely polar strands swell and build an extensive hydration shell in the process which counteracts the close interaction of biomaterial and the surface. B) Owing to the incorporated functionality of the brushes, bacteria are killed when they are in contact with the surface due to the interaction with the extended polycationic segments in low‐salt conditions. It may be conceived that cellular debris accumulates on the surface as a result of contact killing, which shields arriving microorganisms from the contact killing effect. The dynamic properties shown in (A) may allow in the future, although not addressed in this study, for the facile reactivation of such debris‐laden surfaces, by exposure to high salt and rinsing. After the debris has been washed off, the exchange of the solution with a low‐salt environment leads to the collapse of the antifouling moiety, effectively recovering the surface in “killing mode.” Note that the size ratio of proteins, bacteria and polymer does not correspond to the actual ratio.

To harness in the future these effects in the context of salt‐responsive polymer coatings with antibacterial and antifouling properties, the above mentioned properties are combined in a triblock copolymer. The antibacterial polymers are both readily accessible by straightforward synthetic procedures and allow the coating of arbitrary geometries by a simple dip coating and annealing. This concept can be expanded to also include an antifouling moiety, if the difunctional copolymer (antibacterial block + anchor block) is complemented with a polyzwitterionic block. Transferred to the application on a surface, this means that the polymer brush is comprised both of polycationic and polyzwitterionic bristles, whilst the phosphonic acid block roots it to the metal oxide interface (**Figure** **1**). In order to prevent the charged strands from obstructing each other, the anchor block should constitute the central segment.

As mentioned above, utilizing the (anti)polyelectrolyte effect, the expansion of the respective bristles in an aqueous environment can be controlled by varying the ionic strength: in a low‐salt environment, the cationic block stretches far into the solution, while the polyzwitterionic segment is collapsed as it is not soluble under these conditions. Upon addition of salt, the positive charges of the polycation are screened, resulting in a more relaxed conformation. Conversely, the polyzwitterionic moiety now expands and dominates the interface. Since the polymer is firmly attached to the sample, the supernatant solution can be switched without impairing the brush, allowing for numerous transitions between one or the other mode.

## 2 Results and Discussion

The realization of salt‐responsive polymer coatings with antibacterial and antifouling properties rely on the application of new (co)polymers that were first synthesized and fully characterized. Subsequent to the investigation of the adsorption behavior on oxide covered titanium surfaces and a thorough characterization of the individual targeted functions, initial microbiology tests were carried out to support the validity of the contact killing strategy also depicted in Figure 1B.

### 2.1 Synthesis and Characterization of Zwitterionic and Cationic Copolymers

**Scheme 1:**
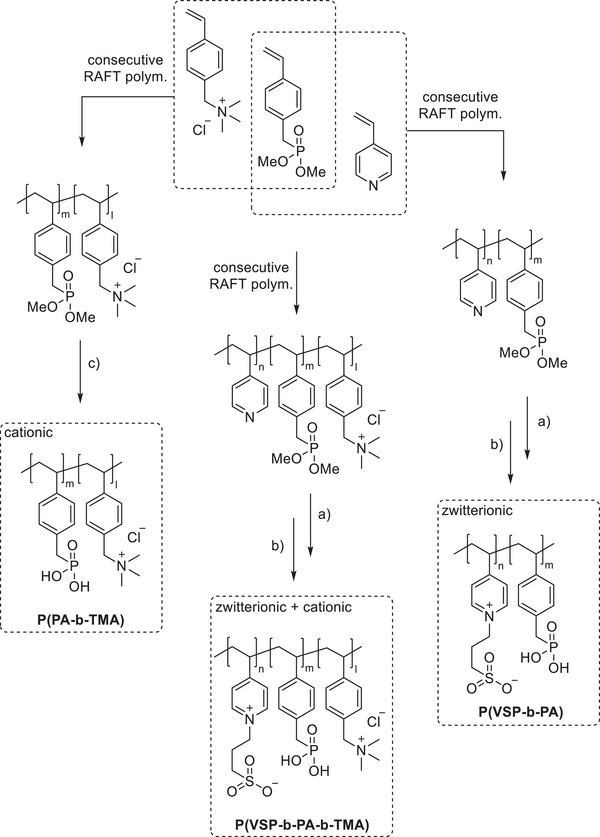
General synthesis of charged di‐ and triblock copolymers containing PA, VSP, and TMA units. Reaction conditions: a) 1,3‐propane sultone (3 eq. per VP unit), HFIP, 40 °C, 3d; b) 6 m HCl/1 m NaCl, 115 °C, 3–26 h; c) 6 m HCl, 115 °C, 26 h.

**Figure 2:**
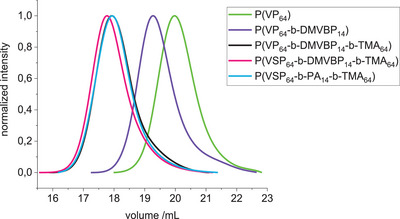
SEC traces of polymers (in HFIP) involved in the synthesis of P(VSP_64_‐*b*‐PA_14_‐*b*‐TMA_64_).

The chain transfer agent 2‐(dodecylthiocarbonothioylthio)‐2‐methylpropionic acid (DMP) and monomers, namely 4‐vinyl pyridine (VP), dimethyl 4‐vinylbenzyl phosphonate (DMVBP), and 4‐vinylbenzyltrimethyl ammonium chloride (TMA), provided the synthetic platform for the responsive triblock copolymer (**Scheme** **1**). Details of the synthesis of the monomers DMVBP and TMA can be found in the Supporting Information. First, P(VP_64_) was obtained by Reversible Addition Fragmentation Chain Transfer (RAFT) polymerization in DMF with 82% monomer conversion and 53% yield. After isolation by precipitation in toluene it was chain extended with DMVBP to yield P(VP_64_‐*b*‐DMVBP_14_) with 44% monomer conversion and 77% yield. Based on the previous results regarding the optimal anchor block length, a degree of polymerization of 14 was expected to ensure good grafting densities without impairing the overall polymer solubility in water later on.^[^ ^43^ ^]^ TMA was then used to form the polycationic block by further chain extension via RAFT polymerization. Since TMA and the derived polymer are not soluble in DMF, water was used as cosolvent. In the final polymer brush, the charged strands should have approximately the same length to ensure that depending on which mode is activated, one dominates the interface while the other is collapsed, hence the degree of polymerization of the TMA block was matched to the VP block. P(VP_64_‐*b*‐DMVBP_14_‐*b*‐TMA_64_) was afforded with 90% (crude) yield and 80% monomer conversion after precipitation in acetone. This did not remove residual monomer completely; however, this was not expected to impair the following reactions and could be easily removed via dialysis afterwards. The polymerization reactions proceeded in a controlled manner as evidenced by monomodal distributions in SEC and dispersities *Ð* of 1.33–1.53 (**Figure** **2**).

In order to introduce zwitterionic groups, pyridine moieties were reacted with 1,3‐propane sultone. This cyclic sulfonate ester is frequently used to prepare betaine structures by nucleophilic ring opening which proceeds quantitatively at elevated temperatures.^[^ ^44^ ^]^ Fluorinated alcohols like hexafluoro isopropanol (HFIP) and trifluoroethanol are suitable solvents for both polyzwitterions and polycations despite their opposing solubility in water with respect to its ionic strength. Thus, the reaction of the triblock precursor with 1,3‐propane sultone was conducted in HFIP at 40 °C and the polymer was isolated by removing roughly half of the solvent in vacuo and dialysis against 1 m NaCl and deionized water. The quantitative formation of the betaines can be verified in ^1^H NMR spectra by the shift of the aromatic proton signals adjacent to the pyridine/pyridinium nitrogen atom from 8.0–8.4 ppm to 8.5–9.1 ppm as well as the emergence of broad resonances at 2.5–2.7, 3.0–3.4, and 4.4–5.0 ppm caused by the sulfopropyl groups. The SEC analysis reveals a shifted monomodal distribution and a slight increase in dispersity to 1.65 (Figure 2). The polyzwitterionic character became evident as the polymer was not soluble in water anymore but readily dissolved upon addition of NaCl. Lastly, the methyl phosphonate groups were converted to the respective acid by acidic hydrolysis in 6 m HCl/0.5 m NaCl. After purification by dialysis and lyophilization, the characteristic shift of the phosphorous resonance in the^[^ ^30^ ^]^ P NMR spectrum from 30.8–32.6 to 18.1–22.4 ppm proved the quantitative liberation of the acid. Once again, the elugram confirmed the monomodal molecular weight distribution and revealed a slight decrease in dispersity to 1.50, which was presumably due to removal of lower molecular weight polymer fractions during dialysis. The final polymer poly(4‐vinyl pyridinium propane sulfonate‐*block*‐4‐vinylbenzyl phosphonic acid‐*block*‐4‐vinylbenzyl trimethylammonium chloride) (P(VSP_64_‐*b*‐PA_14_‐*b*‐TMA_64_)) requires a NaCl concentration of about 0.5 m to be soluble in water owing to the antipolyelectrolyte properties of its zwitterionic block.

To investigate each functionality separately, the respective diblock copolymers containing only the anchor block and either the polycationic or the polyzwitterionic block were synthesized as well (Scheme 1). The general procedure was analogous to the triblock copolymer and afforded the targeted polymers, whose SEC and NMR data are presented in **Table** **1**. All syntheses were conducted on a multigram scale, demonstrating the convenient access to these structures.

**Table 1:** Analytic data for charged di‐ and triblock copolymers.

Polymer	*M*_n_ (NMR) [g mol^−1^]	*M*_n_ (SEC) [g mol^−1^]	*Ð*
P(VSP_63_‐*b*‐PA_13_)	17 300	9400^a)^	1.51^a)^
P(PA_16_‐*b*‐TMA_101_)	24 500	31 000	1.56
P(VSP_64_‐*b*‐PA_14_‐*b*‐TMA_64_)	33 000	27 000	1.50

^a)^ After conversion to phosphonic acid groups, the polymer became insoluble in HFIP, thus data obtained before the hydrolysis of the polymer are given.

### 2.2 Investigation of the Functionality of Polymer Layers

#### 2.2.1 Adsorption on Titanium Oxide Particles

**Figure 3:**
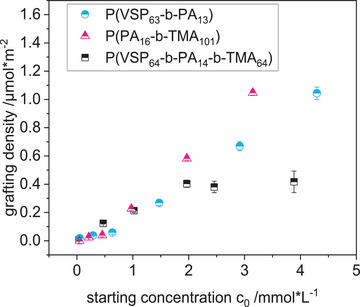
Grafting density of charged di‐ and triblock copolymers on titanium oxide particles at different concentrations of polymers. Solvents: 1 m NaCl for P(VSP_63_‐*b*‐PA_13_), deionized water for P(PA_16_‐*b*‐TMA_101_), 0.5 m NaCl for P(VSP_64_‐*b*‐PA_14_‐*b*‐TMA_64_). The error bars denote the standard deviation (*n* = 3).

The adsorption of the polymers on titanium oxide surfaces was first studied on titanium oxide particles. In particular, the grafting densities were determined for all three charged polymers by determining the residual polymer concentration in solution after adsorption via UV–vis spectroscopy (**Figure** **3**).^[^ ^43^ ^]^ Since the particle radii are larger than typical radii of gyration of the polymers, the effect of surface curvature on adsorption can be neglected. The ionic strength of the aqueous solutions was adjusted to obtain a good solvent for the respective polymer: 1 m NaCl for P(VSP_63_‐*b*‐PA_13_), deionized water for P(PA_16_‐*b*‐TMA_101_), and 0.5 m NaCl for P(VSP_64_‐*b*‐PA_14_‐*b*‐TMA_64_).

Depending on the starting concentration, grafting densities of up to 1 µmol m^−2^ were observed. In the investigated range, the diblock copolymers showed an increase in grafting density with increasing polymer concentration, only P(VSP_64_‐*b*‐PA_14_‐*b*‐TMA_64_) exhibited a saturation plateau. Interestingly, under conditions of lower salt, which leads to a more stretched conformation of the polymers, a dense occupation of the surface was observed. For conventional polyelectrolytes of larger molar mass an opposite effect has been reported.^[^ ^45^ ^]^ The zwitterionic copolymer P(VSP_63_‐*b*‐PA_13_) showed a slightly more efficient adsorption compared to the polycation P(PA_16_‐*b*‐TMA_101_) even though it possesses fewer PA units, which may suggest that the interplay between nonadsorbing block, the solvent and/or block order influence the process. This is emphasized by the comparison with the previously discussed polymers.^[^ ^43^ ^]^ The best performing polymer with 21 PA units showed a similar adsorption isotherm as the diblock copolymers with only 13 and 16 PA units investigated here.

Up to a concentration of about 2 mmol L^−1^ the adsorption isotherm of triblock copolymer P(VSP_64_‐*b*‐PA_14_‐*b*‐TMA_64_) exhibited a similar behavior as the zwitterionic/cationic diblock copolymers. Then, the grafting density plateaued at about 0.4 µmol m^−^^2^, which is a plausible result considering that one equivalent of triblock copolymer effectively formed two bristles, thus taking up more space on the surface than the diblock copolymers. This was exacerbated by the solvent being merely a compromise between the two optimal environments for each strand, namely high ionic strength for the polyzwitterion and low ionic strength for the polycation. Presumably, neither block was expanded as distinctly as it was the case for the diblock copolymers in more optimized conditions. Therefore, the system was likely to be closer to the mushroom regime in comparison, making it hard for oncoming chains to penetrate the already adsorbed polymer bristles.^[^ ^46^ ^]^ Nevertheless, based on the previous investigations regarding poly(4‐vinylpropylpyridinium) (P(VPPr)) based polymers^[^ ^43^ ^]^ and results from literature with similar grafting densities of “grafting to” derived polymer brushes, the surface affinity of the responsive polymer was adequate for the modification of titanium samples.

#### 2.2.2 Adsorption on Planar Titanium Oxide Substrates

The formation of polymer brushes on TiO_2_ terminated surfaces by adsorption of di‐ and triblock copolymers was investigated via surface plasmon resonance (SPR) measurements. The substrates were flat LaSFN9‐glasses coated with chromium (≈1 nm), gold (≈50 nm), and titanium oxide (≈4 nm), obtained by vacuum evaporation. The brush was then prepared by grafting the polymer onto the titanium oxide layer from solution (30 mg mL^−1^; 1 m NaCl for P(VSP_63_‐*b*‐PA_13_), deionized water for P(PA_16_‐*b*‐TMA_101_) and 0.5 m NaCl for P(VSP_64_‐*b*‐PA_14_‐*b*‐TMA_64_), followed by annealing at 120 °C. Subsequently, the substrate was cleaned with 1 m NaCl (for zwitterionic polymers), water and ethanol to remove unbound polymer. The SPR measurements were conducted in a flow cell against deionized water.

**Figure 4:**
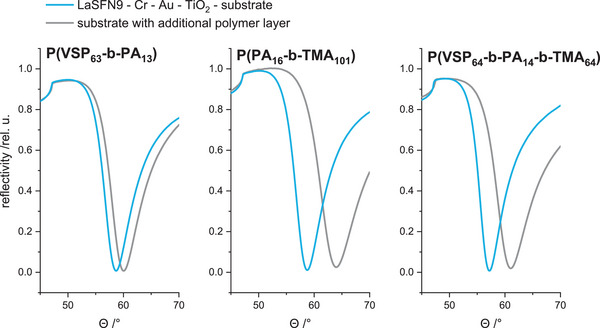
Full SPR angle scans of titanium oxide terminated gold substrates before and after coating with charged di‐ and triblock copolymers against water.

For all polymer coatings, a significant shift of the SPR minimum was detected, which indicates the formation of stable, i.e., irreversibly adsorbed adlayers (**Figure** **4**). Fitting the total reflection edge and the plasmon minimum yielded average (optical) layer thicknesses of 4.4 nm for P(VSP_63_‐*b*‐PA_13_), 8.0 nm for P(PA_16_‐*b*‐TMA_101_) and 5.1 nm for P(VSP_64_‐*b*‐PA_14_‐b‐TMA_64_) (**Table** **2**; Tables S1 and S2, Supporting Information). The refractive index of the polymers was assumed to be 1.58 and 1.62, respectively.

**Table 2:** Polymer layer data from SPR measurements obtained from full angle scans and fitting of total reflection edge and plasmon minimum *Θ*_min_. (Detailed parameters can be found in Tables S1 and S2 in the Supporting Information).

Polymer	Thickness [nm]	*Θ*_min_
P(VSP_63_‐*b*‐PA_13_)	4.4	60.04°
P(PA_16_‐*b*‐TMA_101_)	8.0	63.96°
P(VSP_64_‐*b*‐PA_14_‐*b*‐TMA_64_)	5.1	61.04°

Remarkably, the zwitterionic diblock copolymer yielded layers with the lowest apparent thickness, although a comparable grafting density was to be expected for the chosen concentration of the grafting solution. Considering the solvent, however, the zwitterionic chains likely collapsed in the absence of ions and formed a flat layer rather than a swollen polymer brush. Opposingly, the strands of the cationic diblock copolymer were extended into the supernatant solution, resulting in a thicker layer. The determined values matched the order of magnitude of comparable polymer brushes made via “grafting to”, indicating that the end tethering had worked as hypothesized and unbound polymer was removed during the cleaning process.^[^ ^47^ ^]^

In independent experiments glass substrates were coated with titanium (80 nm) or with titanium (≈2.5 nm), gold (≈50 nm), and again titanium (≈2.5 nm) by evaporation in high vacuum, followed by cleaning in an oxygen plasma to remove any organic surface contaminants. The coating with polymer was carried out from 1 m NaCl for P(VSP_63_‐*b*‐PA_13_) and P(VSP_64_‐*b*‐PA_14_‐*b*‐TMA_64_) and deionized water for P(PA_16_‐*b*‐TMA_101_), respectively. Polymer‐solution covered substrates were placed in an oven at 120 °C for 18 h. Unbound polymer was subsequently removed via sonification in and rinsing with water. For P(VSP_63_‐*b*‐PA_13_) an additional cleaning step in 1 m NaCl was necessary to remove excess polymer (**Table** **3**).

**Table 3:** Values of dry ellipsometric thickness of polymer‐coated titanium oxide samples.

Sample	P(VSP_63_‐*b*‐PA_13_)	P(PA_16_‐*b*‐TMA_101_)	P(VSP_64_‐*b*‐PA_14_‐*b*‐TMA_64_)
Thickness [nm]	4.1 ± 0.3	3.2 ± 0.6	5.3 ± 0.3

In contrast to the determined layer thickness observed by SPR measurements (cf. Table 2), P(PA_16_‐*b*‐TMA_101_) yielded under those conditions the thinnest dry layer with a dry ellipsometric thickness of 3.2 ± 0.6 nm, followed by the zwitterionic diblock copolymer with 4.1 ± 0.3 nm and the triblock copolymer with 5.3 ± 0.3 nm. Another “grafting to” system may be considered for comparison: in hydroxyl‐terminated random P(Sty‐*r*‐MMA) (Sty = styrene, MMA = methyl methacrylate) copolymers, the layer thickness of the grafted polymer brush was observed to depend on the average molar mass of the chains.^[^ ^48^, ^49^ ^]^ The group of Perego found layer thicknesses of 7.1 and 9.0 nm for *M*_n_ = 11 200 and 19 500 g mol^−1^, when they produced the polymer brushes from the melt. In this work, only a concentration of 10 mg mL^−1^ was used to coat the substrates with the charged block copolymers, which is the reason for the comparably thinner layers although the average molar mass exceeds that of the P(Sty‐*r*‐MMA) brushes of Perego et al. The results agree with the conclusion drawn from the adsorption experiments on titanium particles that the concentration is vital for the grafting density, which determines the average layer thickness.

**Figure 5:**
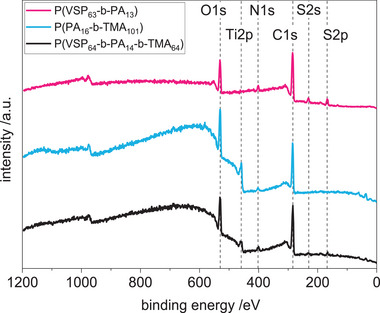
XPS survey spectra of titanium oxide surfaces coated with charged di‐ and triblock copolymers.

The elemental surface composition of the polymer‐coated TiO_2_ terminated substrates was characterized by X‐ray photoelectron spectroscopy (XPS). According to the XPS survey scans, all samples showed O1s, N1s, and C1s peaks at 531, 400, and 285 eV, respectively (**Figure** **5**). Peaks attributed to the S2s and S2p signals at 230 and 167 eV were detected only in the samples modified with zwitterionic segments. High resolution spectra confirmed these results (compare Figure S1 in the Supporting Information). In the C1s spectra, two peaks associated to carbon in different chemical environments were observed at 285.6 and 283.4 eV, respectively, which are attributed to aliphatic carbon atoms in the backbone and alkyl chains and aromatic carbon atoms. The small but discernible P2p peak at 132.0 eV indicates of the presence of phosphorus atoms in all three samples.

The experimental and theoretical atom concentrations were calculated from the XPS data and the structural formulas (**Table** **4**). Overall, the values were in good agreement, with exception of the contribution of nitrogen. Especially for P(PA_16_‐*b*‐TMA_101_), the experimentally determined value was significantly lower than expected, whereas the fraction of oxygen was higher. This may be attributed to partial polymer degradation in the XPS and the increased uncertainty due to correction for the underlying substrate for the thinnest polymer layers.^[^ ^50^ ^]^

**Table 4:** Experimental and theoretical elemental contributions derived from XPS spectra and structural formulas of charged di‐ and triblock copolymers. End groups were neglected in the calculation.

Element	P(VSP_63_‐*b*‐PA_13_)		P(PA_16_‐*b*‐TMA_101_)		P(VSP_64_‐*b*‐PA_14_‐*b*‐TMA_64_)	
	XPS [%]	theor. [%]	XPS [%]	theor. [%]	XPS [%]	theor. [%]
C	71.9	67.1	85.1	86.5	79.3	75.3
O	18.4	20.5	10.7	3.9	13.3	13.1
N	3.6	5.7	1.9	8.3	3.6	7.2
P	0.9	1.2	2.2	1.3	1.3	0.8
S	5.2	5.7	0	0	2.5	3.6

**Figure 6:**
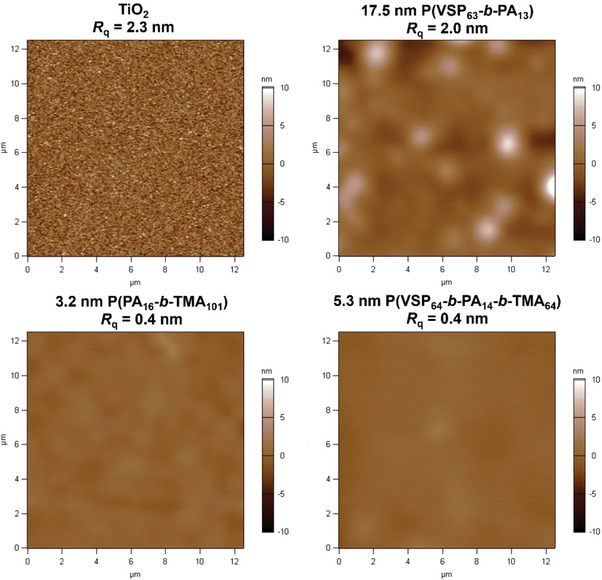
AFM height images of native TiO_2_ and polymer‐coated TiO_2_ substrates. The polymer coated surfaces were washed twice with NaCl solution, except for P(VSP_63_‐b‐PA_13_) which was imaged before the second washing‐step with NaCl solution. This washing step reduced the dry ellipsometric thickness to 4.1 nm.

Furthermore, the surface topography of all samples was investigated using AFM which showed a clear difference in morphology as well as roughness between uncoated TiO_2_ and surfaces modified with P(VSP_64_‐*b*‐PA_14_‐*b*‐TMA_64_) and P(PA_16_‐*b*‐TMA_101_) (**Figure** **6**). Compared to the granular TiO_2_ surface, the polymer layers had a smooth and pinhole free appearance, consistent with a soft, water swellable polymer coating on top of the TiO_2_. The root mean square roughness *R*_q_ decreased from 2.3 nm (TiO_2_) to 0.4 nm for the TMA containing polymer coatings, respectively.

Consistent with the AFM data are the results of SEM analyses of a sample modified with P(VSP_64_‐*b*‐PA_14_‐*b*‐TMA_64_). The flat and homogeneous surface morphology indicates a controlled and consistent coating procedure (Figure S2, Supporting Information).

Static water contact angle measurements revealed a significantly increased wettability of the TiO_2_ surfaces after coating with the charged polymers (**Table** **5**). Similar results were reported for brushes based on polymethacrylates carrying tertiary amine groups and mixed cationic/zwitterionic brushes on titanium surfaces.^[^ ^24^, ^51^ ^]^ P(PA_16_‐*b*‐TMA_101_) lead to the lowest contact angle with 14°, while the zwitterionic diblock and triblock copolymers afforded values of 21° and 29°, respectively. Huang et al. reported similar findings: using salt‐free water, the wettability of zwitterionic surfaces was less pronounced compared to cationic surfaces.^[^ ^24^ ^]^ When the same measurement was conducted with saturated NaCl solution, the wettability of the zwitterionic surface was greatly improved owing to the antipolyelectrolyte effect.

**Table 5:** Static water contact angles of uncoated and coated titanium oxide samples.

Sample	TiO_2_	P(VSP_63_‐*b*‐PA_13_)	P(PA_16_‐*b*‐TMA_101_)	P(VSP_64_‐*b*‐PA_14_‐b‐TMA_64_)
	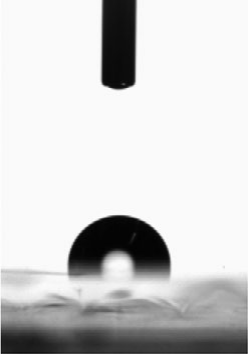	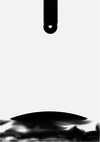	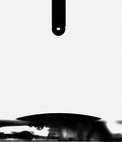	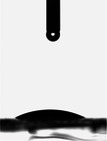
Static water contact angle	94° ± 1°	21° ± 1°	14° ± 1°	29° ± 2°

#### 2.2.3 Protein Adsorption and Salt‐Switching Induced Desorption by SPR

**Figure 7:**
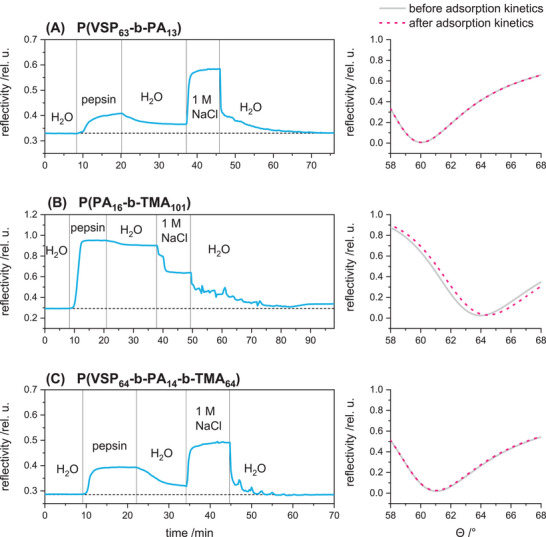
Left: Measurements of reflectivity against time in SPR kinetic mode using deionized water, 1 mg mL^−1^ pepsin in water and 1 m NaCl as solutions in the flow cell with polymer coated substrates at about 1 mL min^−1^. Right: Magnified section of SPR angle scans against water before and after kinetic measurements displaying shifts of the plasmon minimum *Θ*_min_.

In SPR flow experiments, the antiadhesive properties of the coated substrates can be probed by offering a sticky protein and detecting changes in the intensity of the reflected light at a fixed angle.^[^ ^20^ ^]^ An increase in reflectivity corresponds to a shift of the plasmon minimum to higher angles, indicating a thickening or formation of an adlayer. Pepsin, a digesting enzyme, is well‐suited as a model substance for strongly adhering bacterial debris as it is negatively charged due to its low isoelectric point and consequently adsorbs onto surfaces equipped with polycations, similar to cell membrane fragments.^[^ ^52^, ^53^ ^]^ The three substrates were first equilibrated in water and then exposed to a solution of pepsin in water (**Figure** **7**). In all cases, the exposure to the protein solution caused an increase in reflectivity. This is primarily attributed to the adsorption of pepsin onto the polymer layer and to a very minor extent the increase of the refractive index of the medium. The adsorbed protein was only partly removed by subsequently purging the flow cell with deionized water, which is evident since the reflectivity did not reach the starting level. Even the purely zwitterionic surface modification did not resist the adherence of pepsin, which indicates that the presence of ions is necessary for the protective hydration shell to form.

Thus, using a solution containing 1 m NaCl should allow the zwitterionic blocks to swell and release the adsorbed protein in case of (A) P(VSP_63_‐*b*‐PA_13_). Upon exposure for 10 min, the reflectivity increased owing to the higher refractive index of the salt solution compared to water and presumably the extension of the polymer bristles. When changing the environment to water again, the reflectivity dropped to the starting level. The full angle scans before and after the kinetic experiment are congruent, demonstrating that there was neither residual adsorbed peptide nor detachment of the polymer brush (Figure 7). By contrast, the purely cationic P(PA_16_‐*b*‐TMA_101_) brush exhibited a decrease in reflectivity in 1 m NaCl. This can be attributed to shrinking of the adlayer due to a more relaxed conformation of the bristles, whose positive charges were screened by the added ions. When the solution was switched to water again, the reflectivity dropped significantly lower than before the washing step with 1 m NaCl. Although the cationic surface typically does not exhibit a salt dependent antiadhesive effect, the salt solution was more effective in removing adsorbed pepsin than deionized water. Presumably, the ions compete with the adsorbed pepsin due to Coulombic interactions at the charged polymer chains, superseding it from the surface. Nevertheless, the layer thickness surpassed the starting level after the system is equilibrated (>90 min). The full angle scans revealed a shift in the plasmon minimum of 0.5° which corresponded to an average pepsin layer thickness of 3 nm, assuming a refractive index used for fitting of 1.45 (typical values for proteins).^[^ ^54^ ^]^ Similar results have been observed for cationic surface modifications by Lienkamp et al. and demonstrate the underlying problem in the application of contact‐killing implant coatings.^[^ ^20^, ^40^ ^]^ Residual biological matter promotes biofilm formation and shields and thus disables the surface functionality.

Surfaces modified with the triblock copolymer P(VSP_64_‐*b*‐PA_14_‐*b*‐TMA_64_) exhibited the same behavior as the purely zwitterionic modification: after the adsorption of pepsin, water was not enough to remove the protein entirely. The activation of the polyzwitterionic strands was evident by the increase of reflectivity upon flushing with 1 m NaCl, which indicated that these segments were now swollen and expanded. Although the protein was expected to form strong electrostatic interactions with the polycationic segments, the synergy of these segments coiling and the expansion of the heavily hydrated zwitterionic blocks were sufficient to recover the surface completely. This was evident as the starting level was restored and since the full angle scan did not derive significantly from before the adsorption experiment. The increase in reflectivity upon exposure to salt solution demonstrated the selective swelling of the zwitterionic bristles, as the purely cationic diblock copolymer exhibited a decrease in reflectivity in the same conditions. Hence, the combined polymer brush offers the advantage of the antifouling properties, while still incorporating the potentially bactericidal moieties and allows triggering the respective properties by adjusting the ionic strength of the aqueous environment.

#### 2.2.4 Surface Colonization of *S. aureus* and *E. coli*

**Figure 8:**
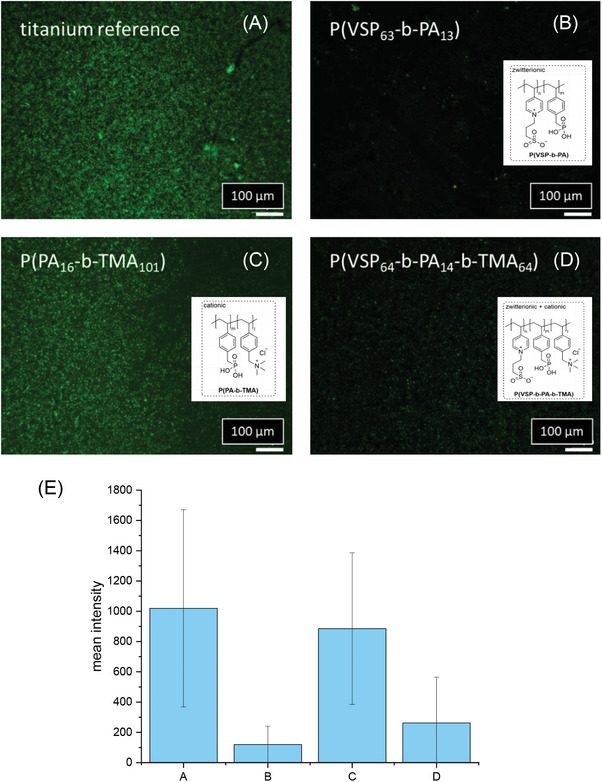
Epifluorescence microscopy images of adherent *S. aureus* on A) uncoated and B–D) coated substrates after incubation with *S. aureus* for 24 h and removal of nonadherent bacteria by rinsing. Green fluorescence (staining with the DNA intercalator Syto9) indicates attached bacteria. E) mean fluorescence intensity of fluorescence pictures (A–D) with error bars indicating standard deviation (A – Ti reference, B – P(VSP_63_‐*b*‐PA_13_), C – P(PA_16_‐*b*‐TMA_101_), D – P(VSP_64_‐*b*‐PA_14_‐*b*‐TMA_64_)).

**Figure 9:**
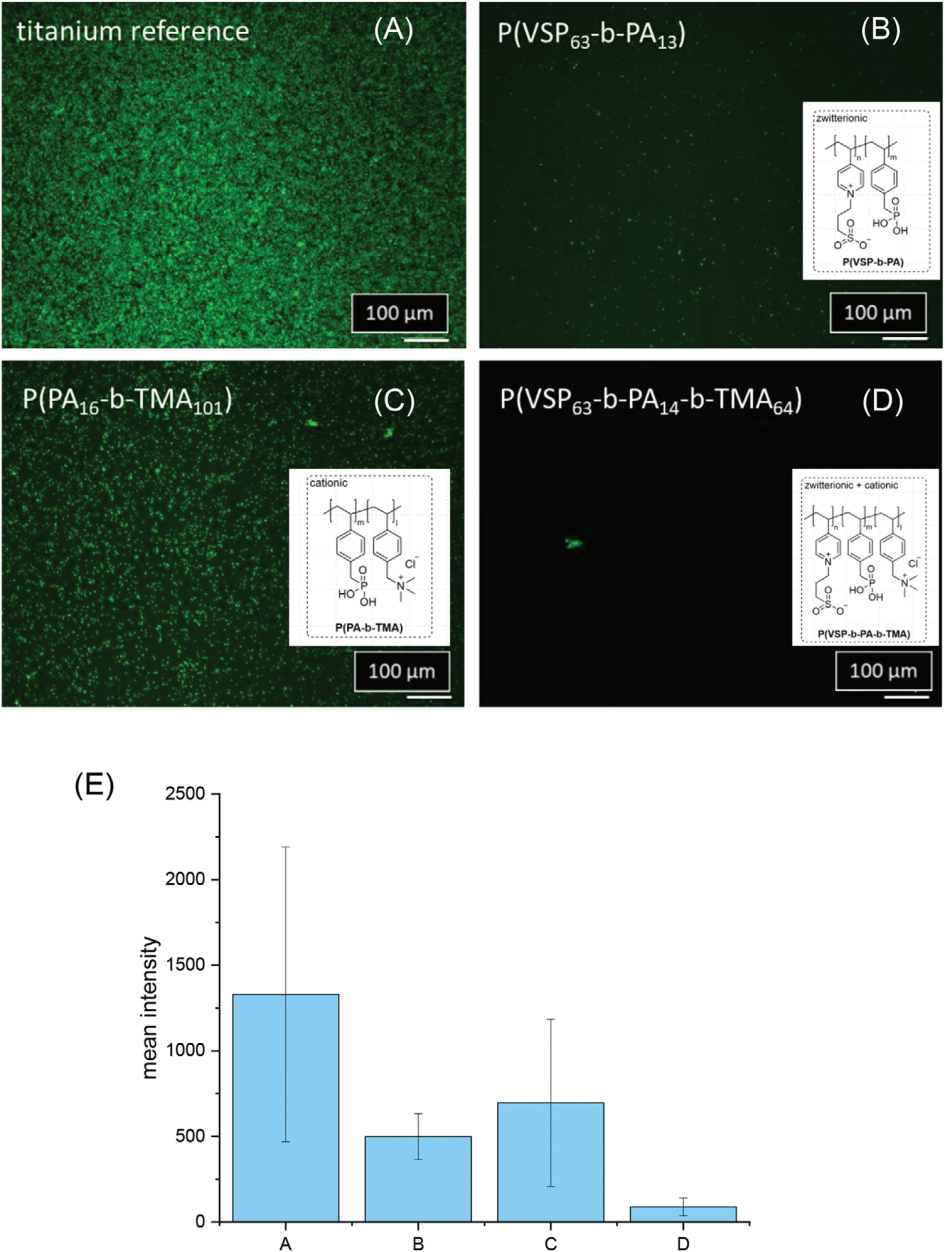
Epifluorescence microscopy images of adherent *E. coli* on A) uncoated and B–D) coated substrates after incubation with *E. coli* for 24 h and removal of non‐adherent bacteria by rinsing. Green fluorescence (staining with the DNA intercalator Syto9) indicates attached bacteria. E) mean fluorescence intensity of fluorescence pictures (A–d) with error bars indicating standard deviation. A – Ti reference, B – P(VSP_63_‐*b*‐PA_13_), C – P(PA_16_‐*b*‐TMA_101_), D – P(VSP_64_‐*b*‐PA_14_‐*b*‐TMA_64_)).

To evaluate the antibacterial effect of polymer coatings with the presented di‐ and triblock copolymers, an uncoated reference substrate and differently coated substrates were incubated for 24 h with both *S. aureus* and *E. coli* as model organisms for Gram‐positive and Gram‐negative bacteria. After bacterial attachment, surface rinsing and fixation with 2.5% glutaraldehyde in PBS, the adhering bacterial derived DNA, including potentially attached extracellular DNA derived from dead bacteria, were stained via a DNA intercalating dye. This was done in order to estimate the surface coverage with bacteria, as DNA is highly concentrated within bacterial cytoplasm. Prior to fluorescence microscopy analysis (**Figures** **8** and **9**), the substrates were rinsed with water.

Both the reference and the surface with exclusively cationic polymers (P(PA_16_‐*b*‐TMA_101_)) exhibited dense coverage with bacteria, while the surfaces modified with the zwitterionic polymers exhibited a low‐fouling profile on large parts of the surface. Here only isolated green spots were discernible, consistent with substantially lower mean fluorescence intensity indicating bacterial coverage. These observations are fully in line with the hypotheses of the strategy depicted in Figure 1.

For the surface occupation of *E. coli* of the titanium substrates coated with P(PA_16_‐*b*‐TMA_101_) and P(VSP_64_‐*b*‐PA_14_‐*b*‐TMA_64_) some areas appeared to be inhomogeneously occupied. Some areas were more densely covered with cells than others, which suggests that the polymer coating was not uniform, considering that the reference sample exhibited a homogenous layer of adhering bacteria. Consequently, the antifouling effect P(VSP_64_‐*b*‐PA_14_‐*b*‐TMA_64_) could presumably be even more pronounced after optimization of the coating process.

It should also be noted that the incubation proceeded in a nutrient solution, which contains about 0.14 mol L^−1^ NaCl among other substances (e.g., yeast extract, Peptone). Thus under these conditions it can be assumed that the zwitterionic chains are swollen to some extent. Since the triblock copolymer also contains positively charged segments, it did not perform as well as P(VSP_63_‐*b*‐PA_13_) for tests with *S. aureus*, but the presence of the zwitterionic block markedly improved its low‐fouling properties compared to P(PA_16_‐*b*‐TMA_101_). Hower, the purely cationic surface modification (P(PA_16_‐*b*‐TMA_101_) exhibited in parts a lower fouling property compared to the reference, especially for *E. coli* (Figure 9A,C). Similar to the results obtained from SPR measurements, these data suggest that the separate effects can be utilized synergistically to combine antibacterial with anti‐ or low‐fouling properties.

#### 2.2.5 Antibacterial Effect in Bulk Bacteria Suspension

The data shown in Figures 8 and 9 refer to stained DNA inside bacteria and potentially also stained extracellular DNA, but cannot provide insight into whether or not the adhering bacteria were still alive. Hence in order to quantify the antibacterial effect of the coatings, classical counting of colony forming units (CFU) was employed. In these experiments the bacteria on the surface as well as bacteria in the medium were analyzed.

**Figure 10:**
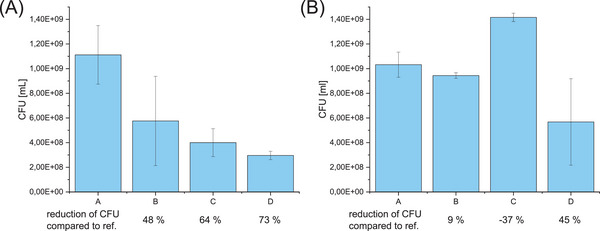
Colony forming units (CFU)/mL and reduction of CFUs compared to reference [%] A) in the *S. aureus* and B) in *E. coli* suspension on top of titanium substrates with and without polymer coating after 24 h incubation. (A – Ti reference, B – P(VSP_63_‐*b*‐PA_13_), C – P(PA_16_‐*b*‐TMA_101_), D – P(VSP_64_‐*b*‐PA_14_‐*b*‐TMA_64_)).

All polymers were observed to lead to a reduction of viable *S. aureus* bacteria compared to the reference sample in the entire 3 mL volume of bacterial suspension on top of polymer coated samples (25 mm × 12 mm) inside microwells (**Figure** **10**A). Although zwitterionic surfaces are not known for their antibacterial properties, a minimal reduction of CFU/mL was observed for P(VSP_63_‐*b*‐PA_13_) as well. The polymers containing polycationic segments lead to a reduction in viable bacteria of 64%, which agrees with the notion that these structures are contact‐active. The presence of zwitterionic groups in case of the triblock copolymer did not interfere with the antibacterial properties of the surface, on the contrary, it exceeded the performance of the purely cationic modification by a small margin.

Since Gram‐negative bacteria possess an inner and an outer membrane, they are generally harder to disrupt by antibacterial polymers.^[^ ^55^ ^]^ Similar to previous results of tests with *E. coli* on a polymer coating with a cationic VPPr block,^[^ ^43^ ^]^ P(PA_16_‐*b*‐TMA_101_) resulted in an increase in CFUs by 37% compared to the reference (Figure 10B). The triblock copolymer containing also a polycationic block, showed for the Gram‐positive and Gram‐negative bacteria tested the most pronounced reduction in CFUs with 73% and 45%, respectively, compared to the reference sample, which suggests that there is a synergy of the zwitterionic and the cationic block which is necessary to impair bacterial viability. The effect of P(VSP_63_‐*b*‐PA_13_) was negligibly small for both *S. aureus* and *E. coli*.

The absolute numbers of the observed effects are comparably small, which is expected since the total number of viable bacteria in the entire volume of 3 mL were analyzed by CFU counting and because the observed reduction stems from contact killing on the surface area of only 3.0 cm^2^. Importantly, the results confirmed the hypotheses regarding the presence of polycationic chains to affect bacterial viability.

Lienkamp et al. reported highly efficient antibacterial coatings of different composition, which showed up to a 6 log reduction against *S. aureus*.^[^ ^20^ ^]^ These coatings are composed of highly crosslinked polymer networks prepared by spin‐coating, resulting in a much denser surface occupation. However, due to the different design of the bacterial assay, the comparability is a priori very limited since Lienkamp et al. used a much smaller volume of bacterial suspension (100 µL vs 3 mL in this work). In order to give a definite evaluation among published systems, the conditions of the respective bacterial assays would have to be reproduced for each efficiency report.

## 3 Conclusion

Three biologically active polymers with anchor segments were synthesized and fully characterized: two diblock copolymers, comprising a polyzwitterionic/antifouling and a polycationic/antibacterial block, respectively. The third polymer combined both of these functional segments with the anchor block in the center. It was demonstrated that the polymers were well‐accessible on a multigram scale via RAFT polymerization with good end group retention and narrow dispersities as confirmed by SEC monitoring. Evaluation with NMR spectroscopy revealed that the subsequent post modifications proceeded quantitatively to afford the targeted polymer structures. Titanium substrates were coated with the charged polymers to afford functional polymer brushes, as confirmed by a large number of surface analytical techniques. The antifouling properties of surfaces with zwitterionic moieties against proteins in presence of salt containing water could be observed in situ via SPR measurements. Most importantly, the irreversible adherence of pepsin (as a model compound for bacterial debris) could still be prevented in case of the triblock copolymer, although it comprised a polycationic segment that favors the adsorption of negatively charged substances. The results confirm the concept of salt‐responsiveness, i.e., the utilization of the (anti)polyelectrolyte effect to switch between blocks. In preliminary microbiological assays, the antibacterial and antifouling properties of coated substrates were probed against *S. aureus* and *E. coli*. Here the synergy of polycationic and polyzwitterionic segment in the triblock copolymer proved most effective and reduced the number of CFUs by 73% and 45%, respectively, compared to a neat oxide covered titanium reference. Staining the adherent bacteria after removal of non‐adherent bacteria uncovered the low‐fouling properties in surfaces equipped with zwitterionic segments (P(VSP_63_‐*b*‐PA_13_) and P(VSP_64_‐*b*‐PA_14_‐*b*‐TMA_64_)), as they significantly impaired surface colonization of bacteria compared to the reference and the surface modified with polycationic chains. Experiments, which give further insight into the salt‐responsiveness in presence of bacteria, recyclability of the surfaces and tests with a library of relevant bacteria strains are next steps in assessing the effectiveness and sustainability of this approach.

## 4 Experimental Section

### Materials

4‐Vinyl pyridine (VP, 95%) was purchased from Merck and distilled before use. Dimethyl‐4‐vinylbenzyl phosphonate (DMVBP) and 4‐vinylbenzyltrimethyl ammonium chloride (TMA) were synthesized according to the literature (see also the Supporting Information).^[^ ^43^ ^]^ AIBN (98%) was purchased from Merck and recrystallized from ethanol before use. Titanium oxide particles (Aeroxide P25), 2‐(dodecylthiocarbonothioylthio)‐2‐methylpropionic acid (DMP, 98%), 1,3‐propane sultone (98%) were purchased from Merck and used as received. 1‐bromopropane and nitromethane were purchased from Acros Organics. DMF (extra dry over molecular sieves) was purchased from Thermo Scientific. Methanol (HPLC grade) was purchased from Carl Roth. Hexafluoroisopropanol (HFIP, 99%) was purchased from Carbolution. Acetone, diethyl ether, isopropanol, and toluene were technical grade solvents. Solvents for NMR analysis (D_2_O, 99.9% and DMSO‐*d6*, 99.8%) were purchased from Deutero.

### Synthesis and Characterization of Polymers

#### Synthesis of P(PA_16_‐b‐TMA_101_)

##### Synthesis of P(DMVBP_12_)


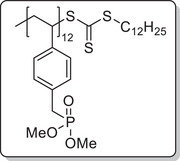


DMVBP (4.1567 mg, 18.38 mmol), DMP (203.1, 0.56 mmol), and AIBN (18.1 mg, 0.11 mmol) were dissolved in DMF (21 mL) in a Schlenk tube under argon atmosphere. The solution was purged with argon for 30 min and placed in a preheated oil bath at 70 °C for 19 h. The reaction was quenched by exposure to air and freezing in liquid nitrogen. For the determination of monomer conversion, a sample was taken and examined by ^1^H NMR spectroscopy. The polymer was isolated by precipitation from cold diethyl ether (300 mL, −50 °C), dissolved in DMF (12 mL) and precipitated again by the same procedure. The polymer was dissolved in ethanol, moved into a flask and dried in vacuo to give the product as a yellow solid (40% conversion, 1.6193 g, 37% yield).

^1^H NMR (DMSO‐*d6*, 500 MHz) δ (ppm): 0.82‐0.87 (br, C_11_H_22_‐C**H**_3_), 1.12‐2.28 (br, C**H**_2_‐C**H** and C_10_ **H**_20_‐CH_3_), 3.03‐3.35 (br, Ar‐C**H**_2_‐P), 3.35‐3.71 (br, O‐C**H**_3_), 4.50‐4.89 (br, S‐C**H**_2_), 6.06‐7.24 (br, Ar‐**H**)

^31^P NMR (DMSO‐*d6*, 202 MHz) δ(ppm): 28.7‐29.7 (br, P)

*M*_n(theo., NMR)_ = 3100 g mol^−1^

SEC (HFIP + 0.05 m CF_3_COOK, calibration with PMMA): *M*_n_ = 1800 g mol^−1^, *D* = 1.19

##### Synthesis of P(DMVBP_12_‐*b*‐TMA_171_)


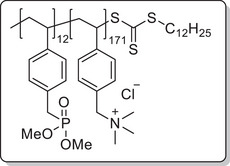


P(DMVBP_12_) (87.9, 0.02 mmol), TMA (1198.6 mg, 5.66 mmol), and AIBN (0.9 mg, 5 µmol) were dissolved in DMF (5 mL) and water (5 mL) in a Schlenk tube with a rubber Septum and a stirring bar. The solution was purged with argon for 20 min and placed in a preheated oil bath at 70 °C afterwards. After stirring for 23 h, the reaction was quenched by freezing the mixture with liquid nitrogen and exposure to air. For the determination of monomer conversion, a sample was taken and examined by ^1^H NMR spectroscopy. The polymer was isolated by dialysis and lyophilization as a colorless solid (94% conversion, 732.4 mg, 57% yield).

Polymers of the general structure P(DMVBP‐*b*‐TMA) can also be isolated by precipitation from isopropanol.

^1^H NMR (D_2_O, 500 MHz) δ (ppm): 0.63‐0.87 (br, C_11_H_22_‐C**H**_3_), 1.05‐2.30 (br, C**H**_2_‐C**H** and C_10_ **H**_20_), 2.74‐3.24 (br, N‐C**H**_3_), 3.51‐3.85 (br, P‐OC**H**_3_), 4.16‐4.66 (br, N‐C**H**_2_‐Ar and P‐C**H**_2_‐Ar), 6.34‐7.61 (br, P‐CH_2_‐Ar**H** and N‐CH_2_Ar**H**)

^31^P NMR (DMSO‐*d6*, 202 MHz) δ (ppm): 31.3‐32.2 (br, P)

*M*_n(theo., NMR)_ = 30 000 g mol^−1^

SEC (HFIP + 0.05 m CF_3_COOK, calibration with PMMA): *M*_n_ = 40 000 g mol^−1^, *D* = 1.44

##### Synthesis of P(PA_16_‐*b*‐TMA_101_)


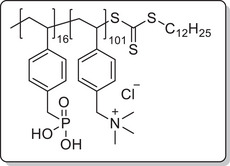


P(DMVBP_16_‐*b*‐TMA_101_) (7 g, 0.28 mmol) were dissolved in water (15 mL) and conc. HCl (15 mL). The mixture was heated to reflux for 3 h, whereby the solution became increasingly turbid. Afterwards, the polymer was isolated via dialysis and lyophilization. The ^31^H NMR spectrum revealed that the conversion to phosphonic acid was not completed, thus the procedure was repeated in water (40 mL) and conc. HCl (40 mL) at 115 °C bath temperature for 23 h, which led to quantitative conversion of the ester. The polymer was purified by dialysis and lyophilization to obtain a colorless solid (4.4698 g, 0.18 mmol, 64%).

^1^H NMR (D_2_O, 500 MHz) δ (ppm): 0.93‐0.98 (br, C_11_H_22_‐C**H**_3_), 1.21‐2.30 (br, C**H**_2_‐C**H** and C_10_ **H**_20_), 2.70‐3.27 (br, N‐C**H**_3_), 4.23‐4.64 (br, N‐C**H**_2_‐Ar and P‐C**H**_2_‐Ar), 6.42‐7.49 (br, P‐CH_2_‐Ar**H** and N‐CH_2_Ar**H**)

^31^P NMR (DMSO‐*d6*, 202 MHz) δ (ppm): 18.3‐21.4 (br, P)

*M*_n(theo., NMR)_ = 24 500 g mol^−1^

SEC (HFIP + 0.05 m CF_3_COOK, calibration with PMMA): *M*_n_ = 31 000 g mol^−1^, *D* = 1.56

#### Synthesis of P(VSP_63_‐b‐PA_13_)

##### Synthesis of P(VP_64_)


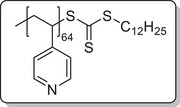


4‐Vinyl pyridine (VP) (3.131 g, 29.8 mmol), DMP (130.1 mg, 0.36 mmol), and

AIBN (8.8 mg, 54 µmol) were dissolved in dry DMF (10 mL) in a Schlenk tube with a rubber septum and a stirring bar. The solution was purged with argon for 30 min and placed in a preheated oil bath at 70 °C afterwards. After stirring for 19 h, the reaction was quenched by freezing the mixture with liquid nitrogen and exposure to air. For the determination of monomer conversion, a sample was taken and examined by ^1^H NMR spectroscopy. The polymer was precipitated from toluene (250 mL), collected by filtration and dried in vacuo, yielding P(VP_64_) (82% monomer conversion, 1724 mg, 53% yield) as red solid.

^1^H NMR (DMSO‐*d6*, 500 MHz) δ (ppm): 0.80‐0.85 (br, C_11_H_22_‐C**H**_3_), 1.17‐1.24 (br, C_10_ **H**_20_), 1.28‐2.25 (br, C**H**_2_‐C**H**), 6.35‐6.96 (br, C_Ar_‐C**H**), 7.99‐8.51 (br, N‐C**H**)

*M*_n(theo., NMR)_ = 7100 g mol^−1^

SEC (HFIP + 0.05 m CF_3_COOK, calibration with PMMA): *M*_n_ = 5100 g mol^−1^, *D* = 1.33

##### Synthesis of P(VP_64_‐*b*‐DMVBP_14_)


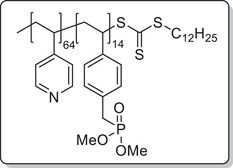


P(VP_64_) (1420.3 mg, 0.19 mmol), DMVBP (1500.2 mg, 6.63 mmol), and AIBN (7.7 mg, 47 µmol) were dissolved in dry DMF (20 mL) in a Schlenk tube with a rubber septum and a stirring bar. The solution was purged with argon for 30 min and placed in a preheated oil bath at 70 °C afterwards. After stirring for 21 h, the reaction was quenched by freezing the mixture with liquid nitrogen and exposure to air. For the determination of monomer conversion, a sample was taken and examined by ^1^H NMR spectroscopy. The polymer was precipitated from cold diethyl ether (250 mL), dissolved in methanol and isolated by removing the solvent in vacuo. P(VP_64_‐*b*‐DMVBP_14_) (44% monomer conversion, 2.24 g, 77% yield) was obtained as a light‐yellow solid.

^1^H NMR (DMSO‐*d6*, 500 MHz) δ (ppm): 0.82‐0.87 (br, C_11_H_22_‐C**H**_3_), 1.18‐1.27 (br, C_10_ **H**_20_), 1.28‐2.25 (br, C**H**_2_‐C**H**), 3.05‐3.27 (br, P‐C**H**_2_), 3.43‐3.63 (br, P‐OC**H**_3_), 6.26‐7.15 (br, PCH_2_‐C_Ar_ **H**‐C_Ar_ **H** and NC_Ar_H‐C_Ar_ **H**), 8.01‐8.45 (br, N‐C_Ar_ **H**)

^31^P NMR (DMSO‐*d6*, 202 MHz) δ(ppm): 29.0‐29.5 (br, P)

*M*_n(theo., NMR)_ = 10 200 g mol^−1^

SEC (HFIP + 0.05 m CF_3_COOK, calibration with PMMA): *M*_n_ = 7900 g mol^−1^, *D* = 1.53

##### Synthesis of P(VSP_63_‐*b*‐DMVBP_13_)


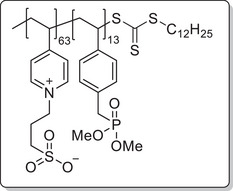


P(VP_63_‐b‐DMVBP_13_) (2.0234 g, 0.2 mmol) and 1,3‐propane sultone (4.6830 g, 38.3 mmol, 3 eq. per pyridine group) were dissolved in HFIP (20 mL) and stirred at 40 °C bath temperature for 22 h. The solvent was removed in vacuo and 0.5 M NaCl (25 mL) was added. The mixture was dialyzed and lyophilized to yield the product as a colorless solid (2.9853 g, 0.17 mmol, 85%).

^1^H NMR (D_2_O+NaCl, 500 MHz) δ (ppm): 1.84‐2.50 (br, C**H**_2_‐C**H** and N‐CH_2_‐C**H**_2_), 3.13‐3.37 (br, P‐C**H**_2_ and S‐C**H**_2_), 3.51‐3.89 (br, P‐OC**H**_3_), 4.77‐5.02 (br, N‐C**H**_2_), 6.82‐8.28 (br, P‐CH_2_‐Ar**H** and N‐CH‐C**H**), 8.64‐9.11 (br, N‐C**H**)

^31^P NMR (D_2_O+NaCl, 283 MHz) δ (ppm): 29.5‐33.2 (br, P)

*M*_n(theo., NMR)_ = 17 700 g mol^−1^

SEC (HFIP + 0.05 m CF_3_COOK, calibration with PMMA): *M*_n_ = 9400 g mol^−1^, *D* = 1.52

##### Synthesis of P(VSP_63_‐*b*‐PA_13_)


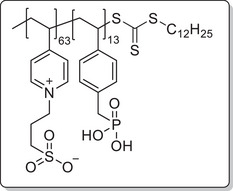


P(VSP_63_‐*b*‐DMVBP_13_) (3.0 g, 0.17 mmol) was dissolved in 1 m NaCl (20 mL) over 6 h. Conc. HCl (10 mL) was added and the mixture was heated to reflux for 3 h. The solution was dialyzed (against 1 m NaCl and distilled water) and lyophilized. Since the ^31^P NMR spectrum revealed that the conversion was not complete, the polymer was again dissolved in 1 m NaCl (20 mL) and conc. HCl (20 mL) and heated to reflux for 23 h. Dialysis and lyophilization of the reaction mixture afforded the product as a brown solid (1.9646 g, 0.11 mmol, 65%).

^1^H NMR (D_2_O+NaCl, 500 MHz) δ (ppm): 1.69‐2.73 (br, C**H**_2_‐C**H** and N‐CH_2_‐C**H**_2_), 2.99‐3.28 (br, P‐C**H**_2_ and S‐C**H**_2_), 4.70‐4.94 (br, N‐C**H**_2_), 6.89‐8.13 (br, P‐CH_2_‐Ar**H** and N‐CH‐C**H**), 8.49‐9.05 (br, N‐C**H**)

^31^P NMR (D_2_O+NaCl, 283 MHz) δ (ppm): 21.7‐25.1 (br, P)

*M*_n(theo., NMR)_ = 17 300 g mol^−1^

SEC (HFIP + 0.05 m CF_3_COOK, calibration with PMMA): the product was not soluble.

#### P(VSP_64_‐b‐PA_14_‐b‐TMA_64_)

##### Synthesis of P(VP_64_‐*b*‐DMVBP_14_‐*b*‐TMA_64_)


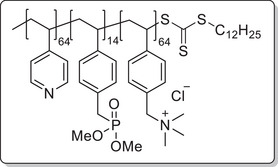


P(VP_64_‐*b*‐DMVBP_14_) (1593.4 mg, 0.15 mmol), TMA (3010.3 mg, 14.2 mmol), and AIBN (5.2 mg, 32 µmol) were dissolved in a mixture of DMF (30 mL) and water (20 mL) in a Schlenk tube with a rubber Septum and a stirring bar. The solution was purged with argon for 30 min and placed in a preheated oil bath at 70 °C afterwards. After stirring for 20 h, the reaction was quenched by freezing the mixture with liquid nitrogen and exposure to air. For the determination of monomer conversion, a sample was taken and examined by ^1^H NMR spectroscopy. The polymer was precipitated from acetone (600 mL), dissolved in methanol and isolated by removing the solvent in vacuo. P(VP_64_‐*b*‐DMVBP_14_‐*b*‐TMA_64_) with contaminations of DMF, methanol, and TMA (4.13 g, 78% monomer conversion) was obtained as a light‐yellow solid. The product was used without further purification.

^1^H NMR (DMSO‐*d6*, 500 MHz) δ (ppm): 0.81‐0.87 (br, C_11_H_22_‐C**H**_3_), 1.16‐1.25 (br, C_10_ **H**_20_), 1.25‐2.31 (br, C**H**_2_‐C**H**), 2.93‐3.30 (br, N‐C**H**_3_ and P‐C**H**_2_), 3.45‐3.63 (br, P‐OC**H**_3_), 4.41‐5.35 (br, (CH_3_)_3_N‐C**H**_2_), 6.12‐7.70 (br, P‐CH_2_‐Ar**H** and N_Pyr_‐CH‐C**H** and N‐CH_2_‐Ar**H**), 8.01‐8.41 (br, N_Pyr_‐C**H**)

^31^P NMR (DMSO‐*d6*, 202 MHz) δ(ppm): 29.1‐29.6 (br, P)

*M*_n(theo., NMR)_ = 24 000 g mol^−1^

SEC (HFIP + 0.05 m CF_3_COOK, calibration with PMMA): *M*_n_ = 23 000 g mol^−1^, *D* = 1.65

##### Synthesis of P(VSP_64_‐*b*‐DMVBP_14_‐*b*‐TMA_64_)


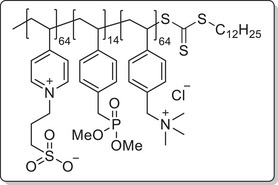


P(VP_64_‐*b*‐DMVBP_14_‐*b*‐TMA_64_) with contaminations of DMF, methanol and TMA (2.985 g) and 1,3‐propanesultone (3.051 g, 24.9 mmol, ≈3.2 eq. per pyridine group) were dissolved in HFIP (21 mL) and placed in a Schlenk flask under argon atmosphere. After stirring at 40 °C for 3 d, roughly ½ of the solvent was removed in vacuo. It was diluted with 15 mL of 1 m NaCl in water and dialyzed against 1 m NaCl in water and deionized water. Lyophilization of the solution afforded the product as colorless solid (2.796 g, 0.08 mmol, ≈71%).

^1^H NMR (D_2_O+NaCl, 500 MHz) δ (ppm): 1.29‐1.36 (br, C_10_ **H**_20_), 1.42‐2.79 (br, C**H**_2_‐C**H** and N‐CH_2_‐C**H**_2_), 3.03‐3.39 (br, N‐C**H**_3_ and P‐C**H**_2_ and S‐C**H**_2_), 3.69‐3.89 (br, P‐OC**H**_3_), 4.44‐5.03 (br, (CH_3_)_3_N‐C**H**_2_ and N_Pyr_‐C**H**_2_), 6.46‐8.22 (br, P‐CH_2_‐Ar**H** and N_Pyr_‐CH‐C**H** and N‐CH_2_‐Ar**H**), 8.49‐9.07 (br, N_Pyr_‐C**H**)

^31^P NMR (D_2_O+NaCl, 202 MHz) δ (ppm): 30.8‐32.6 (br, 1P)

*M*_n(theo., NMR)_ = 33 000 g mol^−1^

SEC (HFIP + 0.05 m CF_3_COOK, calibration with PMMA): *M*_n_ = 30 000 g mol^−1^, *D* = 1.50

##### Synthesis of P(VSP_64_‐*b*‐PA_14_‐*b*‐TMA_64_)


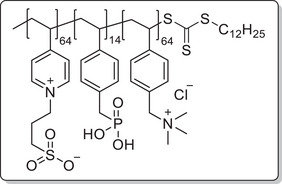


P(VSP_64_‐*b*‐DMVBP_14_‐*b*‐TMA_64_) (2.62 g, 0.08 mmol) and NaCl (10.40 g) were dissolved in 6 m HCl and stirred under reflux for 3 h. The product was isolated by dialyzing the mixture against 1 m NaCl and deionized water. Lyophilization gave P(VSP_64_‐*b*‐PA_14_‐*b*‐TMA_64_) (2.122 g, 0.06 mmol, 81%) as colorless solid.

^1^H NMR (D_2_O+NaCl, 500 MHz) δ (ppm): 1.39‐2.81 (br, C_10_ **H**_20_ and C**H**_2_‐C**H** and N‐CH_2_‐C**H**_2_), 2.81‐3.64 (br, N‐C**H**_3_ and S‐C**H**_2_), 4.38‐5.15 (br, (CH_3_)_3_N‐C**H**_2_ and N_Pyr_‐C**H**_2_), 6.39‐8.41 (br, P‐CH_2_‐**H_Ar_** and N_Pyr_‐CH‐C**H** and N‐CH_2_‐**H_Ar_**), 8.42‐9.10 (br, N_Pyr_‐C**H**)

^31^P NMR (D_2_O+NaCl, 202 MHz) δ (ppm): 18.1‐22.1 (br, P)

*M*_n(theo., NMR)_ = 33 000 g mol^−1^

SEC (HFIP + 0.05 m CF_3_COOK, calibration with PMMA): *M*_n_ = 27 000 g mol^−1^, *D* = 1.50

IR (ATR, ṽ, cm^1^, selected bands): 1038 (versus, S = O), 1184 (versus, S = O), 1475 (m, C‐H), 1641 (m, C = C), 2926 (w, C‐H), 3028 (w, C‐H_Ar_)

###### Instrumentation and Methods

####### NMR Spectroscopy

NMR spectra were recorded on a Bruker (Billerica, MA, USA) Avance 500 and a Bruker Ascent 700 and processed using Bruker TopSpin. The chemical shifts (δ) are listed in ppm and coupling constants (J) are listed in Hz, respectively.

####### Calculation of Molecular Weight

The theoretical molecular weight of the polymers synthesized in this work were calculated using Equation 1 and assuming D = 1. The monomer conversion was determined from 1H NMR samples of the quenched reaction mixture by comparing integrals of monomer and polymer signals

(1) $$M_{n,\;\mathrm{theory}}=\frac{\rho {\left[M\right]}_{0}MW_{M}}{{\left[\mathrm{CTA}\right]}_{0}+2\left[I\right]f}+MW_{\mathrm{CTA}}$$

with *M*_n,theory_ = theoretical molecular weight, *ρ* = monomer conversion, [*M*]_0_ = initial monomer concentration, MW_M_ = molecular weight of the monomer, [CTA]_0_ = initial concentration of CTA, MW_CTA_ = molecular weight of the CTA, [I] = initiator concentration yielding two radicals, *f* = initiator efficiency.

####### Dialysis and Lyophilization

Dialysis was performed with Spectra Pore 6 dialysis membranes (Sigma‐Aldrich, molecular weight cut off 1 kD) against water or as specified in the procedure. An Alpha 2–4 LDplus freeze dryer from Christ (Osterode a.H., Germany) was used to remove water afterwards.

####### IR Spectroscopy

Attenuated total reflection infrared (IR) spectra were recorded on diamond in the range from 370 to 4500 cm^−1^ with a resolution of 4 cm^−1^ on the Bruker (Billerica, MA, USA) “Vertex 70” spectrometer. 16 scans were summed up and processed using ACD/spectrus.

####### Mass Spectrometry (MS)

The samples were analyzed by means of electrospray ionization mass spectrometry (ESI‐MS) using a “Synapt‐G2 HDMS” mass spectrometer from Waters (Manchester, UK) with a time‐of‐flight analyzer.

####### UV–vis Spectroscopy and Adsorption Isotherms

UV/vis spectra were recorded on an Analytik Jena (Jena, Germany) Specord 50 PLUS UV/vis spectrophotometer using Aspect UV software.

To obtain adsorption isotherms, UV/vis‐spectra of solutions in methanol (0.01–0.2 mg mol^−1^) were recorded for calibration for each polymer. Adsorption experiments were conducted by adding the respective amount of polymer and Aeroxide P25 (5 mg) to methanol (1 mL). After treating the samples for 10 min in an ultrasonic bath, they were stirred for 2–4 h. The dispersions were filtered through syringe filters. The resulting solutions were diluted with methanol until the absorption was in the calibrated range and their UV/vis‐spectra recorded to determine the concentration of residual polymer.

####### Size Exclusion Chromatography (SEC)

HFIP + 0.05 m CF_3_COOK as eluent was used in a system with two consecutive columns from PSS (Mainz, Germany) (PSS‐PFG, 10^3^ and 10^2^ Å) and a Merck (Darmstadt, Germany) L‐6200 pump operating at 1 mL min^−1^. A Shodex (Wiesbaden, Germany) RI 101 detector was employed to obtain the molar masses and dispersities according to a PMMA standard.

A second system with THF as eluent and two consecutive columns (PPS‐SDV 10^5^ and 10^3^ Å) and a Merck L‐6200 pump operating at 1 mL min^−1^ with a Knauer (Berlin, Germany) RI detector was employed. The system was calibrated using polystyrene standards.

####### SPR Sample Preparation and Measurement

Surface plasmon resonance (SPR) measurements were performed using a He–Ne laser with a wavelength of 623.8 nm in Kretschmann configuration. A RES‐TEC RT2005 spectrometer (Res‐Tec – Resonant Technologies GmbH, Mainz, Germany) was used with a LaSFN9 prism. LaSFN9 glass wafers were coated via ALD with chromium (≈1 nm), gold (≈50 nm), and titanium oxide (≈4 nm), respectively. For the grafting process, the wafer was overlayed with a solution of polymer overnight. Afterwards, the remaining liquid was removed with a pipette and the sample was dried in a compressed nitrogen flow. It was annealed in an oven at 120 °C for 24 h and thoroughly washed with methanol and absolute ethanol. For kinetic measurements, the angle was set to the flank left to the plasmon minimum at about 30% reflectivity.

####### Atomic Force Microscopy (AFM)

Height images were acquired according to a published method^[^ ^56^ ^]^ under ambient conditions with an MFP‐3D‐Bio AFM (Asylum Research, Santa Barbara, CA, USA) using rectangular silicon cantilevers (Tap300 Al‐G, Budget Sensors, Sofia, Bulgaria) with a nominal resonance frequency of 300 ± 100 kHz, a nominal tip radius of 10 nm, and a nominal spring constant of 40 N m^−1^ in intermittent contact mode. Samples for AFM as well as contact angle, ellipsometry, XPS and SEM analyses were prepared on TiO_2_ coated glass. Elemental Titanium (titanium granules < 6 mm; 99.8%, Chempur, Germany)) was evaporated (Edwards Ltd.E306 coating system, F.D. Edwards, Crawley, UK) onto precleaned glass slides (Menzel Gläser, Braunschweig, Germany) resulting in a thickness of around 80 nm of Ti (Note: nearly identical results were achieved on glass substrates coated with 2.5 nm Ti, 50 nm Au and finally 2.5 nm Ti). Samples were cleaned in an oxygen plasma (Plasma Prep 2, SPI Supplies, West Chester, PA, USA) to remove organic material adhering to the surface and covered with polymer solution (concentration 10 mg mL^−1^ in 1 m aqueous NaCl for P(VSP_64_‐*b*‐PA_14_‐*b*‐TMA_64_) and P(VSP_63_‐*b*‐PA_13_), and pure water for P(PA_16_‐*b*‐TMA_101_)). Substrates were placed in an oven at 120 °C overnight, while the liquid boiled down and evaporated. Afterwards the samples were cleaning by sonication in and rinsing with water, an additional cleaning step in 1 m NaCl solution was applied for P(VSP_63_‐*b*‐PA_13_).

####### Contact Angle Measurements

Static water contact angle measurements were conducted according to the published method^[^ ^57^ ^]^ on an OCA‐15 model instrument (Dataphysics, Filderstadt, Germany) by applying a 2 µL drop of Milli‐Q water to the surfaces. At least three measurements were taken at room temperature of each surface.

####### Ellipsometry

The film thickness of polymers adsorbed to TiO_2_ coated glass surfaces was determined at three different angles (65°, 70°, and 75°) using an alpha‐SE ellipsometer (J. A. Woollam Co., Inc., Lincoln, USA) device with wavelengths between 380 and 900 nm according to a published method.^[^ ^52^ ^]^ The data were fitted with a two‐layer Cauchy model, which contains the background and the polymer layer with the refractive index of 1.50 at a wavelength of 632.8 nm. The first layer was determined as background and the second layer as polymer. Independent measurements for each surface were taken in at least three different regions. The arithmetic mean of the data obtained in the measurements are stated together with the standard deviation (*n* = 3).

####### Tests with Bacteria

LB (Lysogeny broth) was prepared by dissolving 7 g l^−1^ sodium chloride (Th Geyer), 5 g l^−1^ yeast extract (Carl Roth), and 10 g l^−1^ tryptone/peptone (granulated, Carl Roth) in distilled water. Dulbecco's phosphate buffered saline (PBS, 10×, 95 × 10^−3^ m (PO4) DPBS, without calcium or magnesium; Lonza Walkersville, MD USA) was diluted 1:9 in Milli‐Q water (Millipore Elix® Advantage 3, Millipak Filter). 1× PBS and media were autoclaved for 15 min at 121 °C (1.2 bar).

Glycerol stocks of *Staphylococcus aureus* (DSM No. 2569) and *Escherichia coli* NCTC 10 418 stored at −80 °C were streaked with a sterile loop onto LB agar (Luria/Miller, Carl Roth) and incubated at 37 °C. Overnight cultures were prepared by inoculating 3 mL of LB with a single bacterial colony and incubated in a shaker at 180 rpm and 37 °C (incubator MaxQ6000, Fisher Scientific, Hampton, NH, USA) for 17 h ± 2 h. Overnight cultures were adjusted in a cuvette photometer to an optical density (OD600nm) of 0.5 via addition of LB and then 100‐fold diluted in LB. 3 mL of each diluted bacterial suspension corresponding to 2 × 10^7^ CFU (colony forming units)/mL was added into a 6‐well plate (Sarstedt). Each well contained a glass‐Au‐Ti‐Au substrate coated or not coated with the tested polymer, that had been previously submerged in 70% EtOH for sterilization. Plates were incubated for 24 h in a humid chamber at 37 °C without shaking. On the next day the supernatants (floating bacteria) were collected and substrates were washed three times with 3 mL LB medium. Washing solutions were pooled with the supernatant to determine the CFU/mL (concentration of living floating bacteria). For fluorescence microscopic analysis substrates were washed twice with 1× PBS. Next, the cells were fixed in a 2.5% Glutaraldehyde in PBS for 2 h at RT and washed another two times with 1× PBS.

To stain the cells, Syto9 (working solution: 5 × 10^−3^ m, Thermo fisher scientific), that stains bacterial DNA, was diluted 500‐fold in water and 100 µL of the working solution (10 × 10^−6^ m) was pipetted onto parafilm for each condition/substrate and the substrates side incubated with the bacteria suspension on top was placed onto the droplet and incubated at RT for 30 min. Afterwards, the substrates were washed three times with water. Attached bacteria were analyzed with a Zeiss Axio Inverted epifluorescence microscope (Carl Zeiss, Oberkochen, Germany), using a suitable filter set for fluorescence imaging of Syto9 (ex: 450–490 nm, em > 515 nm). Documentation and fluorescence intensity quantification were performed via the software Zen 2012 blue edition version1.1.1.0.

####### Scanning Electron Microscopy (SEM)

Scanning electron microscopy (SEM) was performed with a CamScan 24 (Cambridge Scanning Ltd., Bedford, USA) with an acceleration voltage of 25 kV. To avoid imaging artifacts related to surface charging, samples were sputter‐coated with a thin film of gold (≈30 nm) with a S150B sputter‐coater (Edwards, Crawley, United Kingdom) prior to imaging.

####### X‐Ray Photoelectron Spectroscopy (XPS)

To determine the elemental composition and corresponding concentrations of the polymer‐modified surfaces, an ESCA spectrometer (S‐probe ESCA SSX‐100S Surface Science Instruments, Mountain View, CA, USA) with Al Kα X‐ray radiation of 200 W was used. Analysis of the data obtained was made using Casa XPS processing software (version 2.3.16 PR 1.6). Survey spectra of all elements with an energy resolution 1.0 eV were acquired in the range of 0–1200 eV. Atomic concentrations on the surfaces were determined from the peak areas and sensitivity factors. The experimental values were corrected for the underlying TiO_2_ layer. For high resolution scans, the energy resolution was reduced to 0.1 eV and the spot size was reduced to 300 µm^2^. Prior to fitting the spectra with Casa XPS software, the spectra were shifted adjusting the aliphatic carbon C1s signal to 284.8 eV.

## Conflict of Interest

The authors declare no conflict of interest.

## Supporting information



Supporting Information

## Data Availability

The data that support the findings of this study are available in the supplementary material of this article.
