# Supplementary material for: Salt‐Responsive Switchable Block Copolymer Brushes with Antibacterial and Antifouling Properties
Source: Macromol Biosci. 2024 Nov 27;25(1):2400261. doi: 10.1002/mabi.202400261 (PMC11727821; doi:10.1002/mabi.202400261)
Supplement: Supplementary file 1 — Supporting Information [file MABI-25-2400261-s001.docx]

Supporting Information

Salt-responsive switchable block copolymer brushes with antibacterial and antifouling properties

Rafael Methling, Michael Greiter, Jiwar Al-Zawity, Mareike Müller, Holger Schönherr, Dirk Kuckling*

Synthesis of Monomers

*Dimethyl 4-vinylbenzyl phosphonate (DMVBP)*

4-vinylbenzyl chloride (15 mL, 90.8 mmol) was reacted with trimethyl phosphite (45 mL, 345.0 mmol) with traces of BHT as radical scavenger at 110 °C under argon atmosphere for 19.5 h. Excess phosphite was removed in vacuo and the raw product was isolated via column chromatography (*n*-hexane/ethyl acetate 1:1, then ethyl acetate/acetone 1:1 [R_f_ = 0.47]), which yielded DMVBP as a colorless oil (6.19 g, 27.4 mmol, 30 %).

^1^H NMR (DMSO-*d6*, 700 MHz) δ (ppm): 3.26 (d, ^2^J_PH_=21.7 Hz, 2H, P-C**H**_2_), 3.60 (d, ^3^J_PH_=10.8 Hz, 6H, O-C**H**_3_), 5.24 (dt, ^2^J_HH_=1.0 Hz, ^3^J_HH_=10.9 Hz, 1H, CH=C**H**_trans_), 5.80 (dt, ^2^J_HH_=1.0 Hz, ^3^J_HH_=17.6 Hz, 1H, CH=C**H**_cis_), 6.71 (dd, ^3^J_HH_=10.9 Hz, ^3^J_HH_=17.7 Hz, 1H, Ar-C**H**), 7.24-7.43 (m, 4H, Ar-**H**)

^13^C NMR (DMSO-*d6*, 176 MHz) δ (ppm): 31.4 (Ar-**C**H_2_), 52.8 (O-**C**H_3_), 114.4(CH-**C**H_2_), 126.6 (**C_Ar_**), 130.4 (**C_Ar_**), 132.5 (CH_2_-**C_Ar_**), 136.2 (CH-**C_Ar_**), 136.8 (CH_2_-**C**H)

^31^P NMR (DMSO-*d6*, 202 MHz) δ (ppm): 28.95 (s, P)

ESI-ToF-MS: calculated: 249.0657 g/mol [M+H]^+^, found: 249.0651 g/mol

*4-vinylbenzyltrimethyl ammonium chloride (TMA)*

4-vinylbenzyl chloride (12 mL, 85.2 mmol) was dissolved in ethanol (20 mL) and cooled to 0 °C. Within 20 min, trimethylamine in ethanol (4.2 M, 24.3 mL, 10 mmol) was added. The reaction was allowed to come to room temperature and stirred for 19 h. Ethanol and residual trimethylamine were removed in vacuo. The raw product was recrystallized from acetonitrile to yield TMA (10.27 g, 45.5 mmol, 57 %) as colorless crystals.

^1^H NMR (DMSO-*d6*, 700 MHz) δ (ppm): 3.05 (s, 9H, C**H**_3_), 4.54 (s, 2H, Ar-C**H**_2_), 5.36 (dd, 1H, ^2^J_HH_=0.8 Hz, ^3^J_HH_=10.7 Hz, CH=C**H**_t_**_rans_**), 5.96 (dd, 1H, ^2^J_HH_ =0.8 Hz, ^3^J_HH_ =17.7 Hz, CH=C**H_cis_**), 6.80 (dd, 1H, ^3^JHH =10.9 Hz, ^3^J_HH_=17.7 Hz, C**H**), 7.51 (dd, 2H, Ar-**H**), 7.61 (dd, 2H, Ar-**H**)

^13^C NMR (176 MHz, DMSO-*d*6) δ (ppm): 52.1 (**C**H_3_), 67.7 (Ar-**C**H_2_), 116.2 (CH-**C**H_2_), 126.5 (**C**_Ar_-H), 127.8 (**C**_Ar_), 133.1 (**C**_Ar_-H), 135.8 (Ar-**C**H), 138.9 (**C**_Ar_)

ESI-ToF-MS: calculated: 176.1434 g/mol [M-Cl^-^]^+^, found: 176.1424 g/mol

T_m_ = 260 °C (decomposition)

*Table S1: SPR simulation parameters for substrates coated with P(PA_16_-b-TMA_101_) and pepsin adlayer after kinetic measurements. Pepsin layer thickness depends on the refractive index chosen (= √(EpsX-real)).*

| layer | thickness /nm | EpsX-real | EpsX-imag |
| --- | --- | --- | --- |
| LaSFN9 | 0 | 3.4036 | 0 |
| Cr | 1 | -6.3 | 10 |
| Au | 46.8 | -11.9987 | 1.5976 |
| TiO_2_ | 3.7 | 3.02 | 0 |
| Polymer | 8 | 2.5 | 0 |
| Pepsin adlayer | 0.6 or 7.2 | 2.56 or 1.8225 | 0 |
| Water | 0 | 1,773 | 0 |

*Table S2: SPR simulation parameters for substrates coated with P(VSP_64_-b-PA_14_-b-TMA_64_). No significant adlayer of pepsin was detected after the kinetic adsorption measurements.*

| layer | thickness /nm | EpsX-real | EpsX-imag |
| --- | --- | --- | --- |
| LaSFN9 | 0 | 3.4036 | 0 |
| Cr | 1 | -6.3 | 10 |
| Au | 45.82 | -12.0965 | 1.526 |
| TiO_2_ | 3.4 | 3.35 | 0.268 |
| Polymer | 5.1 | 2.64 | 0 |
| Water | 0 | 1.783 | 0 |


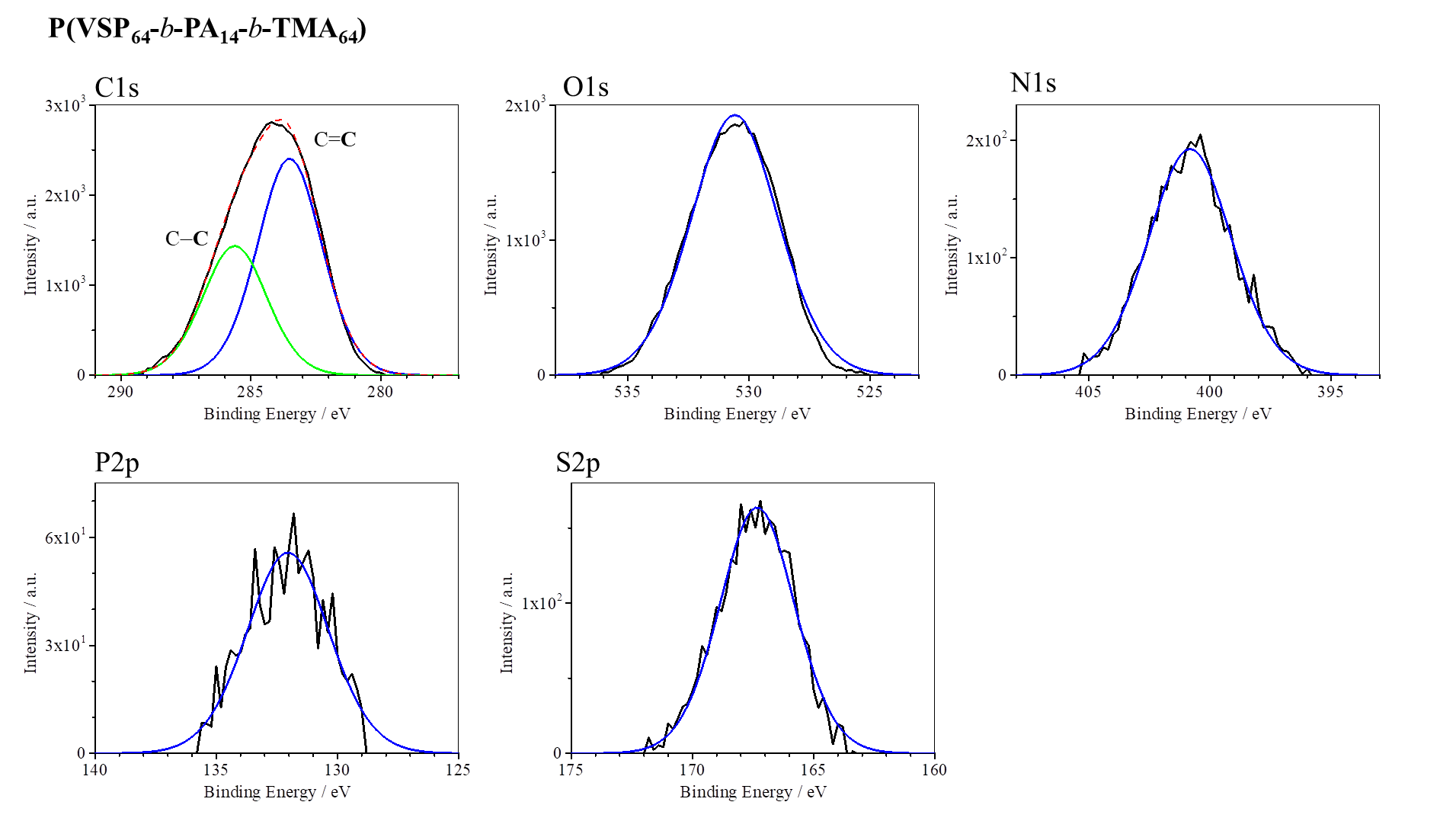


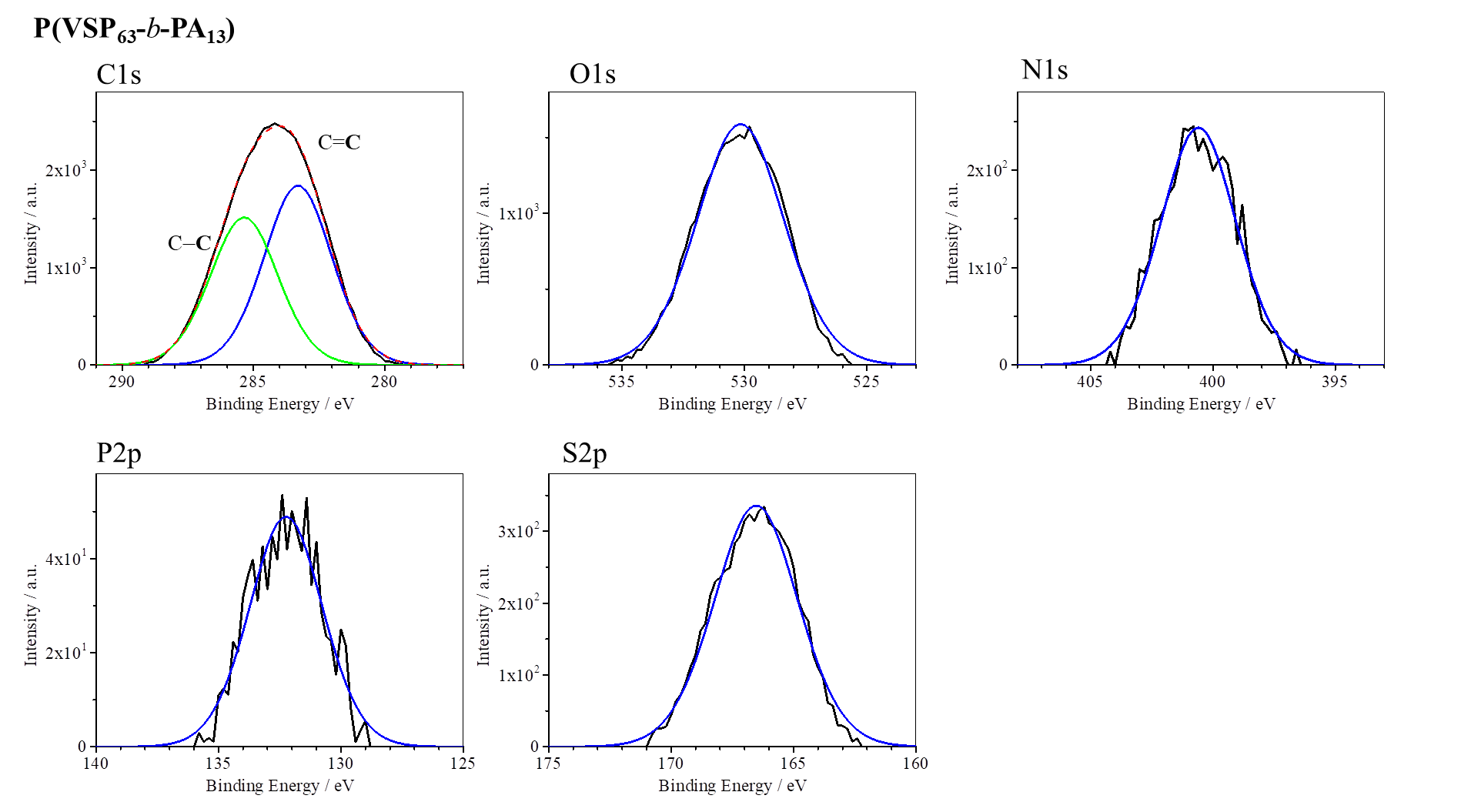


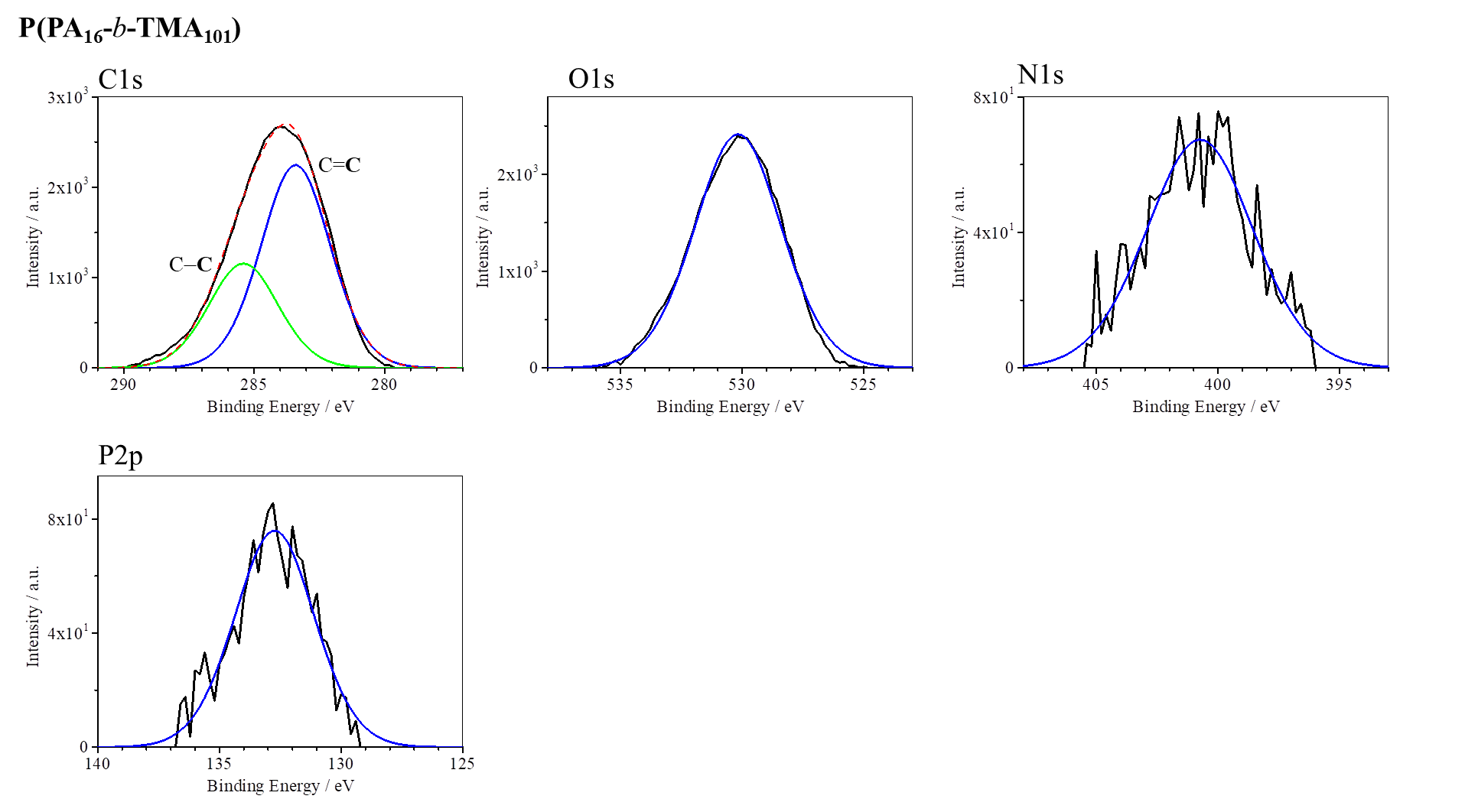


*Figure S1: XPS high resolution spectra.*


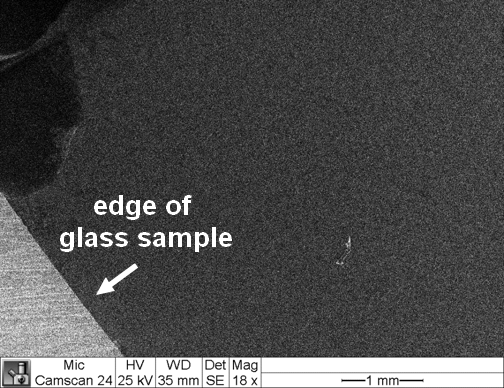









*Figure S2: SEM images of sample coated with P(VSP_64_-b-PA_14_-b-TMA_64_) at different magnifications (18x, 200x, 3000x, 20000x).*
